# Supplementary material for: The user and non-user perspective: Experiences of office workers with long-term access to sit-stand workstations
Source: PLoS One. 2020 Jul 28;15(7):e0236582. doi: 10.1371/journal.pone.0236582 (PMC7386596; doi:10.1371/journal.pone.0236582)
Supplement: S1 File — (DOCX) [file pone.0236582.s002.docx]

**S2. File. Interview guide for all profiles**

**All interviews**

-Introduction researchers and research topic
-Introduction participants (age, gender, work experience and job type will be determined)

*Interactive assignment:*This assignment is used as an ‘ice-breaker’, to get the interview flowing comfortably. Participants are grouped in duo’s and are asked to discuss the following topics with each other and indicate with a post-it on the flip over:
-the most important reason for you to make use of the standing option of the desk.
-the most important reason *not* to make use of the standing option of the desk.

This may result in one or two post-its per category.

**Interview guide employees**-Questions:

- Do you make use of the sit-stand workstation?
  - How frequent and how long?
- What are reasons not to use the sit-stand work stations? (barriers)
- What are reasons to make use of the sit-stand work station? (facilitators)
- Do you know how to use the sit-stand workstations?
  - What is correct height (for sitting and standing)?
  - What are correct body postures?
- What are other actions you take to reduce sitting or increase physical activity (during and outside work)?
  - Or what would be a possible intervention for you in the future?
- In the questionnaire research we conducted in April 2017 here, we found that non-users of the sit-stand workstation believed that because they exercise enough in their free time, they do not need to use the sit-stand workstation (33,6% agree) opposed to users (5,7% agree). Can you reflect on this?
- What would you need to increase the use of your sit-stand desk?
- Are there any other interventions / needs to reduce sitting (at the workplace)
  - Other supportive measures to increase the use of the sit-stand workstation?

**Interview guide Ergo-coaches**-Questions:

- Do you make use of the sit-stand workstation?
  - How frequent and how long?
- What are reasons not to use the sit-stand work stations? (barriers)
- What are reasons to make use of the sit-stand work station? (facilitators)
- Why is the (structural) use if the sit-stand workstation important?
  - In what way are advantages also known by the employees?
- What are main reasons to advise employees to (increase) use of the sit-stand work station?
- What are reasons for some employees not to (start to) use the sit-stand workstations?
  - What can you do to change their minds?
- In the questionnaire research we conducted in April 2017 here, we found that about one third of the employees does not use the sit-stand workstation, one third uses it weekly / monthly and one third uses it on a daily basis. Can you reflect on this?
- In the questionnaire research we conducted in April 2017, we found that most employees think that a training about health promotion in the workplace would be a good intervention to reduce sedentary behaviour at the office, with frequent users of the sit-stand workstation (53,0% agree) a bit more opposed to non-users (36,4% agree). Can you reflect on this?
- If anything was possible, what should be implemented to increase the use of sit-stand workstations?
- If anything was possible, what should be implemented to decrease sitting behaviour in general?

**Interview guide managers**-Questions:

- Do you make use of the sit-stand workstation?
  - How frequent and how long?
- What are reasons not to use the sit-stand work stations? (barriers)
- What are reasons to make use of the sit-stand work station? (facilitators)
- Do you think it is important that employees sit less during their work?
  If yes:
  - Short term (health) effects
  - Long-term health effects

If not:

- - Why?
- In the questionnaire research we conducted in April 2017 here, we found that about one third of the employees does not use the sit-stand workstation, one third uses it weekly / monthly and one third uses it on a daily basis. Can you reflect on this?
- In the questionnaire research we conducted in April 2017 here, we found that frequent users of the sit-stand workstation believed it made them more productive (32,7% agree) opposed to non-users (2,9% agree). Can you reflect on this?
- How would you, as a manager, stimulate the use / increase the use of the sit-stand workstations?
- How would you, as a manager, stimulate the reduction of sitting behaviour in general (inside and outside the workplace)?
- How can the employer / (higher)management play a role in changing (sitting) behaviour of employees?
