# Supplementary material for: The user and non-user perspective: Experiences of office workers with long-term access to sit-stand workstations
Source: PLoS One. 2020 Jul 28;15(7):e0236582. doi: 10.1371/journal.pone.0236582 (PMC7386596; doi:10.1371/journal.pone.0236582)
Supplement: Attachment — Submitted filename: Plos one standing desks_rev_2.docx [file pone.0236582.s003.docx]

PONE-D-20-01975

The user and non-user perspective: experiences of office workers with long-term access to sit-stand workstations

Comments to the author:

I enjoyed reading this interesting study. I think the use of qualitative research methods is a novel approach which provides an in-depth insight into the views and opinions of employees using sit to stand workstations. However, I think that your manuscript could be strengthened by addressing my comments below.

Broad Comments

Introduction:

While the introduction cites important literature in the area of sit-to-stand workstations and sedentary behaviour, in places I felt that it lacked coherence and clarity. I think that in places the lack of coherence detracted from the overall justification for conducting the study. Overall, I think that the justification for conducting this study, using qualitative methods in particular, could be improved. Below is a list of questions I felt were unanswered by the introduction. Consideration of these questions could strengthen the justification for conducting the study:

- Why sit-to-stand workstations as opposed to other forms of sedentary behaviour interventions? Is there evidence to suggest that sit-to-stand workstations are more effective for reducing sedentary behaviour time/ improving health outcomes than other methods?
- Why are qualitative research methods the most appropriate data collection method for examining this specific research question?
- Why is this study novel? What gap is it addressing in the literature?

Methods:

I commend the authors for reporting their study in line with the COREQ guidance.

Although I think most aspects of the methods undertaken have been reporting well, based on the reporting of the thematic analysis conducted in this paper, I do not believe that a reader could follow the analysis steps undertaken, in a future study. To strengthen this section, the authors could report specifically how they undertook each of the six steps of thematic analysis, as outlined by Braun and Clark.

Results:

In places I found the results section difficult to follow. For example the following sentence (line 178): “*Themes within reasons for use and non-use of the standing option were reasons for non-use, reasons for both use and non-use, and reasons for use*”. I think this sentence is referring to sub-themes within the larger theme of “reasons for use and non-use of standing option”. I think that the clarity of the results section could be improved like so: *Themes within “reasons for use and non-use of the standing option” were “reasons for non-use”, “reasons for both use and non-use”, and “reasons for use”.* I think that there needs to be a clear differentiation throughout the results section, to denote where the names of themes or subthemes are referred to. I think that the results section should be updated throughout to reflect this suggestion.

Specific comments:

Introduction:

Page 3 line 48- although I do not speak or understand Dutch, it appears that Reference 8 is a secondary website quoting data from another study. Is this the most appropriate reference for this study? Has the data been published elsewhere? I apologise if this is incorrect. Were the participants from this study Dutch? If so I think this should be clarified here. Can the authors please clarify if “accumulated total sitting time” refers to all sedentary behaviour domains (e.g. sitting at home as well as at work), or just at work?

Page 3, line 48. As far as I can see, reference 11 does not explore the use of sit to stand work stations for reducing sedentary behaviour. Can the authors confirm if the correct reference has been cited here?

Page 4, line 53- please can the authors expanding upon what was included in “multi-component” interventions?

Page 4, line 54- Can the authors please clarify what they mean by “similar effects” here? Do they mean similar to a different type of intervention or similar to the 57 minute reduction in sedentary time reported in reference number 12.

Page 4, line 55- was there any evidence in references 12, 13 and 14 of the variation in reductions in sedentary behaviour? Perhaps standard deviations could be reported to support this claim.

Page 4, line 58- should this line read “between studies” rather than “between individuals”? Please could the authors clarify here whether there were differences in intervention strategies within an individual trial, or whether across a number of trials the strategies were different?

Page 4 line 58, please could the authors provide some more information about the “Stand-up Victoria” study, as it is not mentioned before now.

Second paragraph of the introduction- the authors have reported here a number of studies which examine a range of techniques used to modify sedentary behaviour. But, in my opinion, they have not fully justified why sit to stand work stations may be an appropriate option for reducing sedentary behaviour, and most importantly, whether this reduction in sedentary behaviour improves health or wellbeing outcomes. Please refer to my earlier major comment regarding the introduction of the paper, which I think will help to address this point.

Page 4 line 65- I commend the authors for their discussion of a recent meta-synthesis of qualitative work examining the acceptability and feasibility of sit to stand work stations. However, I felt this important paragraph lacked coherence in places, and I found it difficult to read. I would suggest that the authors restructure the paragraph so that the findings of review are clearly reported. Perhaps the types of studies should be described together and then the findings of the review should be stated, to improve the flow of this paragraph.

Page 4, line 81. Should this sentence perhaps read “previous and current users of sit to stand workstations” instead of “users and ceased users”?

Page 4, line 83- can the authors please clarify here what they mean by “the inclusion of ceased users was a novelty”. Do they mean a novelty in terms of the evidence base?

Page 4 line 85- can the authors please clarify here what they mean by “indicates parallels with the user profile”. What specifically does this indicate is similar between the “user” and “ceased user” groups in reference number 24?

Page 5, line 26. Reference number 26- could the findings of this study be described in more detail in the introduction?

Methods:

Can the authors please confirm how many participants were include in each focus group? I note this information has been included in the results section, but typically this information is reported in the methods section.

Page 5, line 104- Can the authors report here the number of staff within the participating organisation? This will give an indication of the percentage of total staff that were recruited to the study.

Page 5, line 108- I think “group interviews” are typically referred to as focus groups in qualitative literature. I would suggest the authors amend this throughout the manuscript.

Page 7, line 144- please can the authors clarify what they mean by “deliberate debate” which was conducted during focus group schedule development and what this process consisted of. Apologies, I am unfamiliar with the term.

Page 7, line 152- “This warm-up exercise was intended to get familiar with the topic and the group”, can the authors please clarify here if this sentence is referring to the participants themselves, or the researcher?

Page 7, line 157- the authors state here that “*The summaries were used to predefine the major themes for data analysis*”. Was this an evidence based analysis decision? If so, please could the authors cite an appropriate source here? If not, could the authors consider in the limitations section of the discussion if pre-defining themes based on a summary document given to participants could have inhibited the analysis process as outlined by Braun & Clarke?

Page 8 first paragraph. The data analysis process that were undertaken and reported here seem to contradict the process which was reported on line 157. At what point during data analysis was the coding tree developed? Was it developed from the summary sheet given to participants or following thematic analysis process?

Results

Page 8, Line 177- sentence beginning “Furthermore, an extra theme”. Is this sentence complete? It does not make sense as it stands.

Page 8, line 179-80- The sub-theme “reasons for use” is expanded upon here but the other subthemes are not. Is there a rationale for this choice?

Page 8- does the first paragraph of the results section present a summary of the main findings? If so, I think naming this section accordingly would guide the reader more clearly through the results section. Furthermore, the structure of the results section does not match this summary section (the sections are in a different order in the summary and the text). To help the flow of the results section, the summary section should list the themes in the order they are presented in the text, or the result section should be reorganised accordingly.

Page 9- would the participants section and Table 1 not fit better within the methods section?

Page 9, table 1- does the row “Work experience” refer to the length of time the participants have been employed by the organisation where the research was conducted? Or total work experience in any organisation? Please could the authors clarify this in the table by either making the heading clearer, or adding a legend?

Page 9, line 193-194- please can the author clarify what they mean by “hardly ever used”? I think this needs to be more specific.

Page 10, line 225: While interesting, I am not sure that discussion of children being a barrier to leisure time physical activity behaviour is relevant to the research question. I think this should be removed.

Page 11- line 244-45: Can the authors present a quote here to illustrate the participants views about the “health risks” associated with prolonged sitting.

Page 15, Line 343- Please can the authors expanding upon the “several health benefits” reported by participants as reasons for use of the sit to stand work stations? Perhaps an illustrative quote here would help.

Page 16, line 360: please can the authors clarify what they mean by “self-activation”. Is this a term from the literature? I think a definition is required here.

Page 17 line 378- please can the authors confirm who the “several tips” were provided by? Were they provided by the researchers to the participants, or from participant to participant?

Discussion

Page 22, line 499: Please can the authors clarify what they mean by “barely mentioned” here.

Page 23 line 521: can the authors cite any empirical evidence here reporting the “acute benefits” of reducing sedentary behaviour. I think this statement needs to be more specific, and also supported by the evidence base.

Page 23, line 533: can the authors confirm what they mean by “physically dynamic work culture”. I have not read this phrase elsewhere. This also needs to be clarified on line 540.

Page 23, line 535: If reference number 46 finds that standing meeting furniture causes physical and psychological discomfort, why would they be recommend for use here? What alternatives would the authors suggest to facilitate “standing meetings”?

Page 24, line 550. The authors state here that the inclusion of a range of nationalities within the sample is a strength of their study. While I agree, I cannot find information presented in the manuscript about the nationalities of those included. I think this information could be included in or near to Table 1.

Conclusion

Page 25, line 578: How do the authors propose that researchers identify users and non-users of an intervention before an intervention is implemented?
