# Supplementary material for: The user and non-user perspective: Experiences of office workers with long-term access to sit-stand workstations
Source: PLoS One. 2020 Jul 28;15(7):e0236582. doi: 10.1371/journal.pone.0236582 (PMC7386596; doi:10.1371/journal.pone.0236582)
Supplement: Attachment — Submitted filename: Point_by_Point_revisions_V2.docx [file pone.0236582.s004.docx]

**Point-by-point revisions.**

We addressed the comments of both reviewers point-by-point here below. When applicable, the adjusted text from the manuscript is inserted after the point addressed. Line numbers refer to the unmarked manuscript, without track-changes. Emphasis in adjustments are underlined.

REVIEWER 1

1. Thank you for the opportunity to review this interesting piece of work. I think this has a lot of potential, however I would suggest the authors should be encouraged to resubmit a revised version. My main concerns are in relation to the language used which was confusing and made the paper very difficult to read and follow. I wouldn’t be confident it would be written clearly enough to be accessible to non-specialists. I found the language complicated and sentence structure clunky and difficult to navigate. Please consider reworking the grammar, syntax and choose less complicated terms e.g. triangulation, reticence. I think this paper would really benefit from extensive proof reading. I also found the reporting of the results very hard to follow. Please consider a table or figure or some sort of graphic that helps explain the themes, sub themes and codes for the various groups involved. Once this paper is more “reader friendly”, I think it will make a good addition to the literature.

**Response:** Thank you for reviewing our article. We have checked the document throughout on language and adjusted it when needed and many simplifications were made, also according to the suggestion of the reviewers. We have replaced words as reticence and triangulation. Furthermore, we restructured the results section in order to make it clear that a hierarchy exists within the themes: There are two major themes with themes and subthemes and one extra separate theme with subthemes. E.g.: [line 188-197]: *“Two major themes were identified during data analyses: 1) reasons for use and non-use of the standing option and 2) strategies to increase the use of the standing option and physical activity at the workplace. Furthermore, an extra separate theme, “contextual and individual factors”, was identified. Within major theme 1 – reasons for use and non-use of the standing option – the following themes were identified: “reasons for non-use”, “reasons for both use and non-use”, and “reasons for use”. Reasons could lie within personal preferences (i.e. be a conscious choice) or in external causes (i.e. be an unconscious motive). Within major theme 2 – strategies to increase the use of the standing option and physical activity – the following themes were identified: “solutions addressing reasons for non-use of the standing option” and “other strategies for being active at work”.”*

Because of the restructuring of the results-section, making it clear that this hierarchy within the data presentation exists, we feel it is not needed to graphically explain the themes in a separate figure. However, in the three figures with the two major themes and the extra separate theme, we have added the hierarchy now to further clarify this.

2. Sentence line 27-28, please reword as I found it hard to read.

**Response:** We have adjusted the sentence to make the message more clear. See line [28-30].
*“Furthermore, providing information and reminders were mentioned as strategies to increase standing and physical activity. This may lead to a change in the sitting culture within the company, as more employees would adopt active movement behaviours.”*

3. Throughout paper- although I can see why you used the words “acute benefit”, I wonder if immediate or short term, or similar may fit better?

**Response:** Thank you for this suggestion. We have changed this term throughout the paper into *“immediate benefit*”.

4. Line 31-34, here I don’t feel like the conclusion section takes the results described above and makes a summary of them. What are the take-home messages, important findings, implications of the work? To me, this section could be improved.

**Response:** The conclusion has been adjusted now and starts with the most important finding from the results section and linking these results to the recommendations for future research: [line 31-34]: *“Immediate benefits of the use of the standing option – only mentioned by the users – were the most distinctive reason to use sit-stand workstations. Future research should look into how to motivate users and non-users to adopt a practice period in order to perceive these immediate benefits, whether it is linked to sit-stand workstations or other interventions to reduce sitting time.”*

5. Line 37: Typo “advise”
   **Response:** This has been adjusted, thank you.
6. Line 37-38, please include references for other countries. To my knowledge the Canadian, UK and Australian guidelines also suggest this or very similar.
   **Response:** Thank you for pointing this out**.** We have included the other references to guidelines of the UK, Australia and the Netherlands, in order to provide the complete picture. See references 1-4 in the revised manuscript.
7. Line 40- please consider using the term “device based” instead of objective, as I am not sure they really are totally objective!
   **Response:** We have adjusted this term, in accordance to the reviewer [line: 40-41]: *“…a recent meta-analysis including solely studies with device-based measured sitting time…”*
8. Please reword the sentence ending line 41, especially the last few words of the sentence. Perhaps “of” all-cause mortality instead of “on”.
   **Response:** We have adjusted this sentence [line 41]: *“… associations between prolonged sitting time and increased risks of all-cause mortality.”*
9. Line 43, I think this would be strengthened with the addition of other references here. There are many papers which you could refer to strengthen the point. Please search for papers included in the text below, for guidance.

Sedentary activities have been shown to comprise 65-82% of time at work in industrialised countries (Ryan et al., 2011, Parry and Straker, 2013, Ryde et al., 2013, Clemes et al., 2014, Hadgraft et al., 2016 a) with a large proportion (54-77%) of office workers total daily sitting time occurring during their “working day” (Clemes et al., 2014, Kazi et al., 2014, Waters et al., 2016).

**Response:** Thank you for the additional references. We have included new references on sitting at the office as well as for total sitting time, although we refer to research which has indicated times in hours and not percentages for work times and work days [line 41-43]: *“In office workers, sitting time is highly prevalent (9, 10), with almost 5.5 hours of sitting at the work place (Ryde et al 2019; Ryan et al. 2011), accumulating to total sitting times of ten hours per day (11, Smith et al 2015).”*

10. Line 52, do you need the – minus sign here when you have already said reductions of? And again line 54
    **Response:** We agree with the reviewer and have deleted the minus signs.
11. Line 65- 67 please reword/shorten, I found it hard to follow the sentence flow.
    **Response:** We have shortened the sentence to increase the flow [line 67-69]: “A synthesis of qualitative research [25] included qualitative studies among sitting-based office workers which focussed on health problems associated with sitting [26] and possible strategies to reduce occupational sitting [27, 28]*.”*
12. Line 68- Is this providing info/awareness on their sitting time or info/awareness on how to reduce sitting time, or other?#
    **Response:** We have clarified this by adding [line:70-71]: *“…, included providing information and raising awareness about the health risks of prolonged sitting [26, 27].”*
13. Line 73: “which focused on experiences of users of the furniture.” Please reword.
    **Response:** We have reworded it as follows [line:75 ]: *“…which focused on user-experiences of the furniture.”*
14. Line 76-77 “or the novelty of sit-stand workstations in the trials induced curiosity (21) increasing risk for selection bias”. Please reword. Unsure of what is meant here.
    **Response:** We have clarified this sentence in order to show that when only enthusiastic participants are included, this might provide only positive reflections because of selection bias [line 79-83]: *“The novelty of the furniture induced curiosity [29], increasing the risk for selection bias, by only including enthusiastic participants. This may have resulted in more positive reflections towards sit-stand workstations than applicable to the whole population [31], by neglecting the perspective of office workers who did not use the furniture.”*
15. Line 81-90: Please rewrite, the English used is confusing.
    **Response:** We have clarified this sentence [85-87]: “*A recent study which included interviews with users and ceased users of sit-stand workstations, showed that sustained use of the furniture was dependent of personal considerations such as peer support [32].”*
16. Line 92: “companywide” please change to company-wide
    **Response:** This sentence is adjusted according to your next remark and the remark of reviewer 2. Please see our response to your next comment (#17).
17. Line 92 and 93, it is hard to understand which study you are talking about here. Current study versus the referenced study.
    **Response:** Thank you, this was indeed not clear. This has been adjusted now to better describe our previous study to which we referred to (also in accordance to comment #18 of reviewer 2) and this resulted into [line 96-98]: “*In a recent survey study about the use of sit-stand workstations within a large office population (N=1098) with long-term access to the furniture, we identified three user groups: daily users; monthly/weekly users and non-users [34]. Users and non-users from this survey study were invited to participate in the present qualitative study.”*
18. Line 96: “We used triangulations of perspectives”- I find this phrase confusing, perhaps other readers will too?
    **Response:** The term triangulations is regularly used in qualitative studies. However, we agree with the reviewer that this term might be confusing to some readers. This sentence has been clarified by emphasizing on the perspectives and replacing the term “triangulation”, also in line with our response to comment #1 [line: 101-102]: *“We used different perspectives, including the perspective of users and non-users of sit-stand workstations, as well as of managers and employees who were trained as internal ergo-coach.”*
19. Line 97: “who were trained as internal ergo-coach.” Should this be coaches? I am not sure most readers will understand “ergo-coach”. Is there a better term or maybe add a brief explanation?
    **Response:** We have added a description in the method section to explain the term [line 132-133]: *“Internal ergo-coaches are employees who were trained in the ergonomic principles of a healthy workplace and could be consulted by their colleagues about the organisation and set-up of their work(place).”*
20. Line 106: Why 1999?
    **Response:** This is the year the organisation purchased the furniture, which we have clarified [line: 110-112]*: “All (±2000) employees at this worksite of the organisation have had access to electrically adjustable sit-stand workstations since the start of their employment or since 1999, when the furniture was obtained.”*
21. Line 107- plural or singular “workstations”
    **Response:** Thank you for pointing this out, we have adjusted it to singular.
22. Line 108-109, what is the difference between group interviews and focus groups?
    **Response:** In qualitative research these terms are used interchangeably. However, a focus group typically refers to a group conversation with 6-10 participants. Since not all group interviews included 6-10 participants, we found we could not use this terminology within this research and therefore we used the term group interviews, which could refer to all different kinds of group sizes.
23. Line 110- Are the capitalisations used here correct?

**Response:** Thank you for pointing this out, we have adjusted the capitalisation.

24. Line 111- please provide more info on the consent. Was it informed consent?

**Response:** Yes, participants were informed and were able to ask questions before signing. We have added this information to the sentence [line 117-118]: “*All participants gave written informed consent before commencing the interviews.”*

25. Line 117- “primary process of the organisation,”- unsure what this means
    **Response:** To protect the participants’ and organisations privacy, we could not be more specific about this. Still we indicated it is about processes which involves computer work tasks [line: 122-124]: *“Participants were employees involved in the primary work process (involving computer work tasks) of the organisation, supportive personnel, managers or internal ergo-coaches.”*
26. Line 119-122: Over what time frame? The last week? Month? Year?
    **Response:** This was specified by the question in the previous survey study, in which we asked how often employees used the standing option of their desk *in general*, without referring to a specific recall period. This was indicated as the average use. We now also added this term in our manuscript [line 125-127]: “*Of the potential participants 47 had a non-user profile (self-reported average use of the sit-stand workstation of less than once per week), 70 had an user profile (self-reported average use of the sit-stand workstation of at least once per week to several times per day)…”*

27. Line 122- “with different work content,” Unsure what this means
    **Response:** This phrase has been deleted from the sentence as it was double information. The sentence now reads [line: 128-129]: *“Per user-profile, two group interviews were planned, one with employees involved in the primary work process and one with supportive personnel.”*
28. Line 132-134- please reword
    **Response:** This sentence has been reworded into [137-138]: *“In the selection of participants for the groups with specific user- and job profiles, we aimed to maximize variation in participant characteristics, such as gender and age.”*
29. Line 135- is this semi structured? Please mention here if so. Please also be consistent with terminology – interview guide or interview structure (or topic guide)
    **Response:** Thank you for pointing this out. We have now used the term “semi-structured interview guide”, throughout the manuscript [line: 142-143]: *“All members were involved in the development of a semi-structured interview guide.”*
30. Line 144 Please give more information on this process, summarise the decisions made, who made them, on what basis?
    **Response:** We have included a description of the process of the development of the semi-structured interview guide. The draft of the interview guide was based on the qualitative research available to date (such as ref number 27,29 and 30) which was discussed within the research team. We have changed the description of the process to[151-153]: *“The semi-structured interview guide was developed by the research team (see S1 File). A researcher (LR) provided (sub-)questions based on (qualitative) literature to date, which were set in a definitive format after debate within the research team.”*
31. Line 1853- “discussed plenary,” unsure what is meant here
    **Response:** We have substituted the term plenary with a description of the term [line: 162-164]: *“After the exercise, reasons for use and non-use were discussed with the whole group, after which the group interview further evolved.”*
32. Line 167- “resembling the specific management perspective or not”- unsure what this mean
    **Response:** We simplified this sentence and left this part out, which resulted in the following sentence [line: 177-178]: “*Additional codes were discussed and consensus was sought again between coders.”*
33. Line 168- 170: “Triangulations of perspectives from six different user and work profiles, did not require saturation to be met, still for reasons for use and non-use, saturation seemed to be established.” Unsure what this means
    **Response:** We added a phrase to clarify this. There were no new codes identified after all transcripts were coded, which indicates saturation is met [line 183-185]: *“Still, for reasons for use and non-use, saturation seemed to be established, since no new codes were identified during coding of the last parts of the final transcript..”*
34. Please review and re write the data analysis section. This section needs further information.
    **Response:** We rewrote this section and included a more pronounced description of the steps we took in line with Braun and Clark (2006), also in accordance with comment #4 of reviewer 2 [line:169-185]: *Data were analysed using ATLAS.ti 8 software and included three data coders (LR, MH and ES). After data collection, the six steps of thematic (content) analysis were used [36] to identify themes, which involved open coding, and identifying, reviewing and defining (major) themes. After reading of the transcripts to get familiar with the data, open coding of the four user and non-user interview transcripts was done by LR. For one interview transcript (non-user profile, supportive personnel), open coding was also conducted by MH and (general) codes between both coders were discussed. When differences occurred, consensus between the coders was sought. Additional codes from the coding of the other transcripts by LR, were discussed with MH. Open coding of the management interview transcript was both done by ES and LR. Additional codes were discussed and consensus was sought again between coders. The resulting initial coding tree was discussed between LR and HRP. Within the coding tree, two major themes and one extra separate theme with subthemes and main codes were reviewed and identified. This was again discussed within the whole research team and resulted in three conceptual frameworks in which the hierarchy of the two major themes and the extra separate theme were presented. The inclusion of perspectives of six different user and work profiles, did not require saturation to be met. Still, for reasons for use and non-use, saturation seemed to be established, since no new codes were identified during coding of the last parts of the final transcript.”*
35. “derived from the data.” Please re-word
    **Response:** This sentence was re-written into [line: 190-191]: *“Furthermore, an extra separate theme, “contextual and individual factors”, was identified.”*
36. Sentence line 178- Please reword- very confusing
    **Response:** We have tried to make it more clear by adding parentheses (as suggested by reviewer 2 in comment #5) and reworded the sentence into: [line 191-193]: *“Within major theme 1 – reasons for use and non-use of the standing option – the following themes were identified: “reasons for non-use”, “reasons for both use and non-use”, and “reasons for use”.”*
37. Consider adding a table which outlines the themes clearly. I know the figures have the themes in more detail, but perhaps one just identifying all the themes. I also think the figures are very complex and considering there are 3 of them, it may be a lot for the reader to process
    **Response:** Thank you for this suggestion. As stated earlier in this rebuttal (see our response to your comment #1), we have added clarity on the hierarchy of the major themes, themes and subthemes, including the addition of the description of the hierarchy within the figures (1-3). We feel that we now guide the reader through the results section and that all the three figures are vital for this guidance. Adding an extra table would probably increase complexity. Please consider our adjustments in the manuscript and figures based on your first comment.
38. Line 183- unsure what represented means here
    **Response:** This phrase was rewritten into [ line”197-198]: *“Reasons for use and non-use were thoroughly discussed during the interviews, and the manager and internal ergo-coach perspective seemed similar to the user perspective.”*
39. Line 189- is combined the best word to use here?
    **Response:** We have changed this by deleting the word combined [line: 202-203]: *“In table 1, an overview of characteristics is provided for users, non-users and managers and the internal ergo-coaches.”*
40. Line 203-204, please watch syntax here
    **Response:** We have adjusted this sentence, to make it more clear, with consistent terminology [line: 217-219]: *“Subthemes and main codes within the extra separate theme “contextual and individual factors” are presented in figure 1, which included: cultural context, organisational context, interpersonal context and individual attitudes and behaviour.”*
41. Line 204- what theme?

**Response:** We have rewritten this into subthemes as “theme” was the wrong term here [line 219]: *“These subthemes provide a deeper…”*

42. Line 204-207- unclear
    **Response:** We have clarified the sentences to explain that there were no differences between user perspectives mentioned whitin these sub-themes [line: 219-222]:*“* *These subthemes provided a deeper understanding of the background of the population as a whole and within this organisation regarding the concepts of sedentary behaviour and physical activity. There seemed to be no major differences in contextual and individual factors that were mentioned by users and non-users.”*
43. Line212-213- sentence structure makes it very hard to read
    **Response:** We have simplified this sentence and changed it into [line 227-228]: *“Participants – who were from several European nationalities – acknowledged the cycling culture (e.g. using a bicycle for transportation) which is present in Dutch society.”*
44. Line 225-226- would are more relevant example to occupational sitting be better here?
    **Response:** We have deleted the section about activities with children, since this was indeed distracting from the message. This section now only includes the occupational example [line 239-242]: *“Within the interpersonal context, information was provided on the influence of others on being physically active. For example colleagues were mentioned, who could influence (health) perceptions and behaviour at the workplace”*
45. Line 227- what is a direct colleague?
    **Response:** direct colleagues here means the colleagues you have a direct line to, with whom you work together with. However, the influence might come from all colleagues, so we have deleted the term “direct” as suggested by the reviewer (see our answer to comment #44).
46. Line 290- “Work content is leading for working posture”- unsure what this means
    **Response:** We have adjusted this (and accordingly within the whole manuscript) into [line: 304]: *“Work content determines working posture”*
47. Line 343- remove the word “a”.
    **Response:** Thank you, this word has been deleted now.
48. Line 345- what does superficially mean here?
    **Response:** We have clarified this and have deleted the term superficially, which resulted in the following [line 358-360]**:** *“Better blood circulation was mentioned as a reason for use but without referring to reductions of long-term health risks such as cardiovascular disease.”*
49. Line 351- what does profoundly mean here?
    **Response:** Since the term superficially was removed, we have also removed the term profoundly, which made these sentences easier to read [line: 365]: *“Avoiding musculoskeletal complaints were mentioned, including the actual experience…”*
50. Line 410- what kind of standing aids?
    **Response:** standing aids can be used for supported standing, these include high chairs and stools. We clarified this sentence as follows[line: 422-423]: *“…changes to facilitate physical activity such as the implementation of desk bikes, treadmill desks or standing aids such as standing stools.”*
51. Typo end of line 442
    **Response:** Thank you, this word has been corrected.
52. Discussion: please mention how these people had access to some form of prompting software and how you think that may impact findings.
    **Response:** In the results section, the access to rest-break software was described as part of the organisational context [line 238-239]: *“Furthermore, several facilities were offered to the employees, ranging from computer rest-break software to numerous sport facilities.”*

Furthermore, this software was mentioned as a possibility to use as a reminder, as described in the results section addressing reasons for non-use [line: 401-402]: *“Reminders on a daily basis, for example from the rest-break software already available at personal computers, were mentioned to help to maintain (new) routines.”*

53. Discussion: I found that this section had too much repetition of results and not enough discussion of, and comparison with a wider breath of literature. I feel that there could be a section on recommendations for future studies or interventions etc, as it is it is heavily dispersed throughout. Little critique of this other studies in this field when discussing.
    **Response:** The discussion section is rewritten, to reduce repetitions of results and improve the English language. Furthermore, we have placed all recommendations for practice within section ‘4.3 Implications for practice’ which we renamed [line 522]: “4.3 Recommendations for practice”.
54. Apologies if I missed this, what language were the interviews conducted in? If not in English, how were they translated etc?
    **Response:** Thank you for pointing this out. The interviews were held in English, this has been added to the manuscript [line 158 – 159]: *“Interviews were held in English and were audio recorded and transcribed verbatim (by DS).”*

REVIEWER 2.

Comments to the author:
I enjoyed reading this interesting study. I think the use of qualitative research methods is a novel approach which provides an in-depth insight into the views and opinions of employees using sit to stand workstations. However, I think that your manuscript could be strengthened by addressing my comments below.
**Response:** Thank you for revising our manuscript so thoroughly. We have addressed your points here below. Line numbers refer to the unmarked manuscript, without tracked-changes and in the revisions we underlined the text is emphasis were needed.

Broad Comments

Introduction:
While the introduction cites important literature in the area of sit-to-stand workstations and sedentary behaviour, in places I felt that it lacked coherence and clarity. I think that in places the lack of coherence detracted from the overall justification for conducting the study. Overall, I think that the justification for conducting this study, using qualitative methods in particular, could be improved. Below is a list of questions I felt were unanswered by the introduction. Consideration of these questions could strengthen the justification for conducting the study:

1. Why sit-to-stand workstations as opposed to other forms of sedentary behaviour interventions? Is there evidence to suggest that sit-to-stand workstations are more effective for reducing sedentary behaviour time/ improving health outcomes than other methods?
   **Response:** Implementation of sit-stand workstations is very common, opposed to other interventions to reduce sitting time in office workers such as desk bikes, which are (perceived) as affecting the work performance. We stated this now in the introduction [line: 56-57]: *“Furthermore, the use of sit-stand workstations seemed not to affect work performance, opposed to cycling or walking desks (Dupont et al. 2019).”*

Furthermore, the first paragraph has been adjusted as well, which also underlines this point [line: 24-48]: *“sit-stand workstations, have become increasingly prevalent and extensively evaluated [16]. From a public health perspective, this gain in popularity might be promising, since breaking up sitting time could reduce health risk of prolonged sitting in people who are inactive [17].”*

2. Why are qualitative research methods the most appropriate data collection method for examining this specific research question?
   **Response:** We showed in our introduction there are many quantitative studies which show that interventions can be effective to reduce sitting in office workers. To answer the question why or how, qualitative measurements are required and we have added the following to the introduction: [line: 62-65]*: “To increase the reach of interventions, it is important to know why office workers adopt the use of the standing option of their sit-stand workstations and why others do not and whether other strategies are being used to reduce sitting. To understand the why, qualitative research seems most suitable (Cypresse 2015).”*
3. Why is this study novel? What gap is it addressing in the literature?
   **Response:** In addition to the changes that we made in line with your comments above, we have also stressed that the research gap was the non-user profile which was not presented in any of the studies up-to-date [line 78-83]: “*However, participants in these studies did not have experience with interventions such as sit-stand workstations, or sit-stand workstations were implemented shortly before evaluations. The novelty of the furniture induced curiosity [29], increasing the risk for selection bias, by only including enthusiastic participants. This may have resulted in more positive reflections towards sit-stand workstations than applicable to the whole population [31], by neglecting the perspective of office workers who did not use the furniture.”*

Furthermore we have adjusted [line: 91-94]: *“It would be interesting to also include the perspective of non-users of interventions, such as sit-stand workstations, who have had access to the furniture at their workplace, without any reason to request it in the past. In this way, a profound understanding of both reasons for use and non-use of sit-stand workstations could be established.”*

Methods:

4. I commend the authors for reporting their study in line with the COREQ guidance.

Although I think most aspects of the methods undertaken have been reporting well, based on the reporting of the thematic analysis conducted in this paper, I do not believe that a reader could follow the analysis steps undertaken, in a future study. To strengthen this section, the authors could report specifically how they undertook each of the six steps of thematic analysis, as outlined by Braun and Clark.
**Response**: We rewrote this section and included a more pronounced description of the steps we took in line with Braun and Clark (2006) [line:169-185]: *“Data were analysed using ATLAS.ti 8 software and included three data coders (LR, MH and ES). After data collection, the six steps of thematic (content) analysis were used [36] to identify themes, which involved open coding, and identifying, reviewing and defining (major) themes. After reading of the transcripts to get familiar with the data, open coding of the four user and non-user interview transcripts was done by LR. For one interview transcript (non-user profile, supportive personnel), open coding was also conducted by MH and (general) codes between both coders were discussed. When differences occurred, consensus between the coders was sought. Additional codes from the coding of the other transcripts by LR, were discussed with MH. Open coding of the management interview transcript was both done by ES and LR. Additional codes were discussed and consensus was sought again between coders. The resulting initial coding tree was discussed between LR and HRP. Within the coding tree, two major themes and one extra separate theme with subthemes and main codes were reviewed and identified. This was again discussed within the whole research team and resulted in three conceptual frameworks in which the hierarchy of the two major themes and the extra separate theme were presented. The inclusion of perspectives of six different user and work profiles, did not require saturation to be met. Still, for reasons for use and non-use, saturation seemed to be established, since no new codes were identified during coding of the last parts of the final transcript.”*

Results:

5. In places I found the results section difficult to follow. For example the following sentence (line 178): “Themes within reasons for use and non-use of the standing option were reasons for non-use, reasons for both use and non-use, and reasons for use”. I think this sentence is referring to subthemes within the larger theme of “reasons for use and non-use of standing option”. I think that the clarity of the results section could be improved like so: Themes within “reasons for use and non-use of the standing option” were “reasons for non-use”, “reasons for both use and non-use”, and “reasons for use”. I think that there needs to be a clear differentiation throughout the results section, to denote where the names of themes or subthemes are referred to. I think that the results section should be updated throughout to reflect this suggestion.
   **Response:** Thank you for this valuable comment**.** We have restructured the results section in order to make it clear that a hierarchy exists including major themes, themes and subthemes, which we have also addressed in response to comment #1 of reviewer 1. For example we adjusted the following [line: 188-193]: *“Two major themes were identified during data analyses: 1) reasons for use and non-use of the standing option and 2) strategies to increase the use of the standing option and physical activity at the workplace. Furthermore, an extra separate theme, “contextual and individual factors”, was identified. Within major theme 1 – reasons for use and non-use of the standing option – the following themes were identified: “reasons for non-use”, “reasons for both use and non-use”, and “reasons for use”.”*

Specific comments:

Introduction:

6. Page 3 line 48- although I do not speak or understand Dutch, it appears that Reference 8 is a secondary website quoting data from another study. Is this the most appropriate reference for this study? Has the data been published elsewhere? I apologise if this is incorrect. Were the participants from this study Dutch? If so I think this should be clarified here. Can the authors please clarify if “accumulated total sitting time” refers to all sedentary behaviour domains (e.g. sitting at home as well as at work), or just at work?
   **Response:** The website referred to is a Dutch government website, which referred to numbers of their own research. To clarify, we have added an international reference (Smith et al 2015, #14) which showed the same high sitting times in office workers. Furthermore we clarified this sentence and included sitting at work as well [41-43]: *“In office workers, prolonged sitting is highly prevalent [9, 10], with almost 5.5 hours of sitting at the work place [11, 12], accumulating to total sitting times of ten hours per day [13, 14].”*

Also, the participants were from an international organisation which was based in The Netherlands. Therefore, the interviews were held in English, which we have added to the methods section [line: 158-159]: *“Interviews were held in English and were audio recorded and transcribed verbatim (by DS).”*

7. Page 3, line 48. As far as I can see, reference 11 does not explore the use of sit to stand work stations for reducing sedentary behaviour. Can the authors confirm if the correct reference has been cited here?
   **Response:** Thank you for pointing this out. The point we wanted to make here was that interrupting sitting could be beneficial for health at the long-term, for example by using the sit-stand workstation. We have adjusted the phrasing as follows to better match it with the reference [line: 46-48]: *“From a public health perspective, this gain in popularity might be promising, since breaking up sitting time could reduce health risk of prolonged sitting in people who are inactive [17].”*
8. Page 4, line 53- please can the authors expanding upon what was included in “multi-component” interventions?
   **Response:** We have added examples of these component in the text, please see our response to your next comment.
9. Page 4, line 54- Can the authors please clarify what they mean by “similar effects” here? Do they mean similar to a different type of intervention or similar to the 57 minute reduction in sedentary time reported in reference number 12.
   **Response:** We have changed the wording to “similar reductions” in order to link it to the referred reductions of 57 minutes (reference #18) [line 52-55]: “*Randomised controlled trials, conducted in large office populations, introducing multi-component interventions (combining several components, for example coaching, reminders and sit-stand workstations) such as the Stand-Up Victoria study, showed similar reductions at 12 months of around 45 minutes …”*
10. Page 4, line 55- was there any evidence in references 12, 13 and 14 of the variation in reductions in sedentary behaviour? Perhaps standard deviations could be reported to support this claim.
    **Response:** We have deleted this whole sentence, to maintain the flow in this paragraph. We added a sentence before (see also response to your comment #1) about the rationale behind the use of sit-stand workstations (which seem not to affect work performance). This made this sentence unnecessary.
11. Page 4, line 58- should this line read “between studies” rather than “between individuals”? Please could the authors clarify here whether there were differences in intervention strategies within an individual trial, or whether across a number of trials the strategies were different?
    **Response:** In this sentence we referred to the number of strategies mentioned by participants within the Stand-up Victoria trial, which were numerous. We have clarified this as follows [line 57-59]: *“Remarkably, it seemed that actual strategies to reduce sitting time by replacing it with standing or physical movement in the office, are numerous. For the Stand-Up Victoria study, 82 separate strategies were identified by the participants [22].”*
12. Page 4 line 58, please could the authors provide some more information about the “Stand-up Victoria” study, as it is not mentioned before now.
    **Response:** We have clarified this and included the Stand-up Victoria study as an example in the sentence about the multi-component interventions, please see our response to your comment #9.
13. Second paragraph of the introduction- the authors have reported here a number of studies which examine a range of techniques used to modify sedentary behaviour. But, in my opinion, they have not fully justified why sit to stand work stations may be an appropriate option for reducing sedentary behaviour, and most importantly, whether this reduction in sedentary behaviour improves health or wellbeing outcomes. Please refer to my earlier major comment regarding the introduction of the paper, which I think will help to address this point.
    **Response:** Please see our response to your comment #1 about the introduction in which we addressed the (possible) benefits of sit-stand workstations.
14. Page 4 line 65- I commend the authors for their discussion of a recent meta-synthesis of qualitative work examining the acceptability and feasibility of sit to stand work stations. However, I felt this important paragraph lacked coherence in places, and I found it difficult to read. I would suggest that the authors restructure the paragraph so that the findings of review are clearly reported. Perhaps the types of studies should be described together and then the findings of the review should be stated, to improve the flow of this paragraph.
    **Response:** We have improved this paragraph by making clear that the review included studies on sitting-based office workers and studies which were conducted after implementation of sit-stand workstations. This now reads as follows [line 67-77]: *A synthesis of qualitative research [25] included qualitative studies among sitting-based office workers which focussed on health problems associated with sitting [26] and possible strategies to reduce occupational sitting [27, 28]. Potential motivators to reduce sitting time, indicated in the studies among these sitting-based office workers, included providing information and raising awareness about the health risks of prolonged sitting [26, 27]. Musculoskeletal health seemed to be more important as motivator to reduce sitting time than the reduction of long-term health risks, such as diabetes or cardiovascular disease [26]. The synthesis also included studies conducted shortly after implementation of sit-stand workstations [29], alongside natural experiments [30], or during randomised controlled trials [31], which focused on user-experiences of the furniture. Overall, the authors of the synthesis identified support from co-workers and managers as an overall facilitator and a predominant sitting culture as an overall barrier to reduce occupational sitting time [25].”*
15. Page 4, line 81. Should this sentence perhaps read “previous and current users of sit to stand workstations” instead of “users and ceased users”?
    **Response:** Here we have used the wording users and ceased users in line with the authors of the paper we referred to. However, we recognise that these terms could be more clear as suggested by the reviewer and have adjusted the terms [line: 85-87]: *“A recent study which included interviews with current users and previous users of sit-stand workstations, showed that sustained use of the furniture was dependent of personal considerations such as peer support [32].”*
16. Page 4, line 83- can the authors please clarify here what they mean by “the inclusion of ceased users was a novelty”. Do they mean a novelty in terms of the evidence base?
    **Response:** The novelty was indeed the inclusion of the perspective of the previous user. We have added the explanation about this research gap in the last sentence of the paragraph before now [line 81-83]: *“This may have resulted in more positive reflections towards sit-stand workstations than applicable to the whole population [31], neglecting the perspective of office workers who did not use the furniture.”*
17. Page 4 line 85- can the authors please clarify here what they mean by “indicates parallels with the user profile”. What specifically does this indicate is similar between the “user” and “ceased user” groups in reference number 24?
    **Response:** Thank you for point this out**.** These previous users were enthusiastic about the sit-stand workstations, since they requested for it in the past. This is now described as follows [87-89]: *“…the previous users had all specifically requested for a sit-stand workstation in the past, indicating similar levels of enthusiasm as the users, at least in the past.”*
18. Page 5, line 26. Reference number 26- could the findings of this study be described in more detail in the introduction?
    **Response:** We have elaborated on our previous survey-study now [line 96-98]: “*In a recent survey study about the use of sit-stand workstations within a large office population (N=1098) with long-term access to the furniture, we identified three user groups: daily users; monthly/weekly users and non-users [34].”*
19. Methods:
    Can the authors please confirm how many participants were include in each focus group? I note this information has been included in the results section, but typically this information is reported in the methods section.
    **Response:** We have added this information in the paragraph on design and setting [line 114-115]: “*In total, six group interviews were held with two to nine participants, which took about 1.5 hours each, between April and June 2018.”*
20. Page 5, line 104- Can the authors report here the number of staff within the participating organisation? This will give an indication of the percentage of total staff that were recruited to the study.
    **Response:** As suggested earlier by this reviewer (comment#18), we have elaborated on our previous survey study in the introduction, in which we have also indicated the number of participants of the survey study within the whole organisation (N=1098). We also included a number of employees working at the site in the Netherlands now [line: 110]: *“All (±2000) employees at this worksite of the organisation…”*
21. Page 5, line 108- I think “group interviews” are typically referred to as focus groups in qualitative literature. I would suggest the authors amend this throughout the manuscript.
    **Response:** Thank you for this suggestions. In qualitative research these terms are used interchangeably. However, a focus groups typically refers to a group conversation with 6-10 participants. Since not all group interviews included 6-10 participants, we found we could not use this terminology within this research and therefore we used the term group interviews which could refer to all different group sizes.
22. Page 7, line 144- please can the authors clarify what they mean by “deliberate debate” which was conducted during focus group schedule development and what this process consisted of. Apologies, I am unfamiliar with the term.
    **Response:** We clarified this process, also in accordance with a comment (#30) of reviewer 1. We included a description of the process of the development of the semi-structured interview guide [line: 151-153]: *“The semi-structured interview guide was developed by the research team (see S1 File). A researcher (LR) provided (sub-)questions based on the literature to date, which were set in the definitive format after debate within the research team.”*
23. Page 7, line 152- “This warm-up exercise was intended to get familiar with the topic and the group”, can the authors please clarify here if this sentence is referring to the participants themselves, or the researcher?
    **Response:** Thank you, we now indicated that this was an warm-up exercise for the participants [line:161-162]: *“This warm-up exercise for participants was intended to get familiar with the topic and the group.”*
24. Page 7, line 157- the authors state here that “The summaries were used to predefine the major themes for data analysis”. Was this an evidence based analysis decision? If so, please could the authors cite an appropriate source here? If not, could the authors consider in the limitations section of the discussion if pre-defining themes based on a summary document given to participants could have inhibited the analysis process as outlined by Braun & Clarke?
    **Response:** The two major themes were somewhat clear because of the aim of this research: “The aim of this study was to gain insight in reasons for use and non-use of sit-stand workstations as well as insight in other strategies to decrease sitting and increase physical activity in the workplace.” This was not necessarily clear after summarizing. We now deleted this sentence because it is confusing. Furthermore, we have elaborated on our analyses and rewrote paragraph 2.5 “data analysis”. The thematic analyses resulted in an extra separate theme (“contextual and individual factors”) and the two major themes only came up after the thematic coding process. Furthermore, all themes and subthemes were identified by coding. However, because of the clear research questions we had, this may have influenced the coding process, which we have now stated in the discussion section [line 575-579]: *“Because of the clear research aim (to gain insight in reasons for use and non-use of sit-stand workstations as well as insight in other strategies to decrease sitting and increase physical activity in the workplace), the coding process might have been influenced beforehand. However, all themes within the two major themes, and the extra separate theme, resulted from the data analysis process.”*
25. Page 8 first paragraph. The data analysis process that were undertaken and reported here seem to contradict the process which was reported on line 157. At what point during data analysis was the coding tree developed? Was it developed from the summary sheet given to participants or following thematic analysis process?
    **Response:** Please see our response to your previous comment (#24). Furthermore, we have altered the data analysis paragraph and deleted this sentence. Please also see the adjusted analysis paragraph: [line:169-185]: *“Data were analysed using ATLAS.ti 8 software and included three data coders (LR, MH and ES). After data collection, the six steps of thematic (content) analysis were used [36] to identify themes, which involved open coding, and identifying, reviewing and defining (major) themes. After reading of the transcripts to get familiar with the data, open coding of the four user and non-user interview transcripts was done by LR. For one interview transcript (non-user profile, supportive personnel), open coding was also conducted by MH and (general) codes between both coders were discussed. When differences occurred, consensus between the coders was sought. Additional codes from the coding of the other transcripts by LR, were discussed with MH. Open coding of the management interview transcript was both done by ES and LR. Additional codes were discussed and consensus was sought again between coders. The resulting initial coding tree was discussed between LR and HRP. Within the coding tree, two major themes and one extra separate theme with subthemes and main codes were reviewed and identified. This was again discussed within the whole research team and resulted in three conceptual frameworks in which the hierarchy of the two major themes and the extra separate theme were presented. The inclusion of perspectives of six different user and work profiles, did not require saturation to be met. Still, for reasons for use and non-use, saturation seemed to be established, since no new codes were identified during coding of the last parts of the final transcript.”*
26. Results
    Page 8, Line 177- sentence beginning “Furthermore, an extra theme”. Is this sentence complete? It does not make sense as it stands.
    **Response:** We have rewritten this whole section to clarify the structure of major themes and themes and also this sentence was altered [line187-188]: “*Furthermore, an extra separate theme, “contextual and individual factors”, was identified.”*
27. Page 8, line 179-80- The subtheme “reasons for use” is expanded upon here but the other subthemes are not. Is there a rationale for this choice?
    **Response:** We have clarified this section by making the distinction more clear between major themes, themes and subthemes, which we have explained in response to your comment (#3). “Reason for use” is indicated as a theme now, not as a subtheme.
28. Page 8- does the first paragraph of the results section present a summary of the main findings? If so, I think naming this section accordingly would guide the reader more clearly through the results section. Furthermore, the structure of the results section does not match this summary section (the sections are in a different order in the summary and the text). To help the flow of the results section, the summary section should list the themes in the order they are presented in the text, or the results section should be reorganised accordingly.
    **Response:** With the adjustments we have made according to the previous comments of the reviewers we believe we have made the structure of the results section more clear. Furthermore, we now have clarified the two sections on the major themes: [line 267]: 3.3 *Major theme: Reasons for use and non-use of the standing option”* and [Line 382]: *“3.4 Major theme: Strategies to increase the use of the standing option and physical activity”*
29. Page 9- would the participants section and Table 1 not fit better within the methods section?
    **Response:** Thank you for this suggestion, we have been contemplating about this with the co-authors as well. However, since we present results about the participants we felt it was better to include these results in the results section. Still, if the editor and/or reviewer feel it is more suitable in the method section, we are open to change this.
30. Page 9, table 1- does the row “Work experience” refer to the length of time the participants have been employed by the organisation where the research was conducted? Or total work experience in any organisation? Please could the authors clarify this in the table by either making the heading clearer, or adding a legend?
    **Response:** We have specified this in the text [line: 206-207]:*“In general, participants were highly educated, had long working experience at the organisation and worked full-time.”*
31. Page 9, line 193-194- please can the author clarify what they mean by “hardly ever used”? I think this needs to be more specific.
    **Response:** This was specified in table 1 [line 211]. Furthermore, we have now clarified this in the text [line: 207-208]: “*In the non-user groups, participants used the standing option never or less than once per month, except for one, who indicated to use it less than once per week.”*
32. Page 10, line 225: While interesting, I am not sure that discussion of children being a barrier to leisure time physical activity behaviour is relevant to the research question. I think this should be removed.
    **Response:** Thank you for this comment. Also according to a comment made by reviewer 1 (comment #44), we have deleted this section as it was indeed distracting from the core message.
33. Page 11- line 244-45: Can the authors present a quote here to illustrate the participants views about the “health risks” associated with prolonged sitting.
    **Response:** We included the following quote at the end of the paragraph [line 258-260]: “*I think it is quite this kind of common message. Everybody sits too much, everybody eats too much. And we should get more active and standing is more active than sitting.”*
34. Page 15, Line 343- Please can the authors expanding upon the “several health benefits” reported by participants as reasons for use of the sit to stand work stations? Perhaps an illustrative quote here would help.
    **Response:** We have deleted the word ‘several’, as the benefits were mentioned superficially or indirect, such as better blood circulation. This now reads as follows [line 357-360]: *“Health benefits were mentioned by both users and non-users as to why it is or would be better to reduce sitting time and to use the standing option.* *Better blood circulation was mentioned as a reason for use but without referring to reductions of long-term health risks such as cardiovascular disease.”

    Also,* the example about the increased blood circulation becomes clear from the quote at line 361-362: *“Well it is the alternative for getting some movement and improving the blood circulation in your legs ‘cause you get stiff from sitting. So I feel it, I can feel it.”*
35. Page 16, line 360: please can the authors clarify what they mean by “self-activation”. Is this a term from the literature? I think a definition is required here.
    **Response:** Thank you for pointing this out, we have clarified this term as follows [line: 374-375]: *“This reason to use the standing option was specified by self-activation (a way to activate oneself when feeling drowsy) and staying alert during work (when a drop in concentration was experienced).”*
36. Page 17 line 378- please can the authors confirm who the “several tips” were provided by? Were they provided by the researchers to the participants, or from participant to participant?
    **Response:** In the sentence leading to these tips we made it clear now that this was stated by the users [line: 391-393]: “*To address the habitual reasons for non-use, it was stated by the users that the use of the standing option should be incorporated within daily routines. To achieve that, several tips were provided, mostly aimed at a personal change in habits*.”
37. Discussion
    Page 22, line 499: Please can the authors clarify what they mean by “barely mentioned” here.
    **Response:** We tried to explain that the participants did not mention productivity specifically during the interviews. We have now clarified that these statements about productivity were not mentioned directly as such [line: 505-508]: *“In the current study, statements about productivity (gains or losses) were not mentioned as such. Still, high-focus work tasks (e.g. typing), which were mentioned being preferably done while sitting, might indirectly mirror a decrease in perceived productivity if done standing.”*
38. Page 23 line 521: can the authors cite any empirical evidence here reporting the “acute benefits” of reducing sedentary behaviour. I think this statement needs to be more specific, and also supported by the evidence base.
    **Response:** This statement is based on our current study results in which the users mentioned immediate benefits (we changed this term according to comment #3 of reviewer 1) as an important reason for use. We have clarified this now as follows [line: 524-527]: *“When developing interventions, including sit-stand workstations (environmental restructuring), persuasion to adopt a practice period might lead to immediate (experienced) benefits, as indicated by the users in our study, possibly resulting in long-term use.”*
39. Page 23, line 533: can the authors confirm what they mean by “physically dynamic work culture”. I have not read this phrase elsewhere. This also needs to be clarified on line 540.
    **Response:** We have changed this term into “physically active work culture”, which a is more familiar term in general. It now reads [line: 541-544]: *This might become more evident as a transition can be made from a sitting culture towards a more physically active work culture, in which the organisational culture promotes health behaviour [23, 45]. Standing (group) meetings, including the proper furniture, might be included as a strategy to alter the sitting norm towards a more active culture.”*

And [line: 549-550]: *“Interventions to reduce sitting at the office should include shared components, such as incorporating a more physical active work culture,…”*

40. Page 23, line 535: If reference number 46 finds that standing meeting furniture causes physical and psychological discomfort, why would they be recommend for use here? What alternatives would the authors suggest to facilitate “standing meetings”?
    **Response:** In order to overcome these experienced feelings of discomfort, again, a practice period might be needed. We have added this to the sentence now [line: 544-547]: *“Still, there might be more needed than just placing standing tables in a meeting room to overcome the sitting culture, as these might also introduce physical and psychological discomfort [51] and again a practice period might be needed.”*
41. Page 24, line 550. The authors state here that the inclusion of a range of nationalities within the sample is a strength of their study. While I agree, I cannot find information presented in the manuscript about the nationalities of those included. I think this information could be included in or near to Table 1.
    **Response:** We have stated this in the method section. But because of privacy reasons (for the company as well as the participants), no further specifications on the nationalities were made [line: 109-110]: *“Office workers from a large international semi-governmental organisation in The Netherlands were included.”*

Also we included this in the results section now [line: 227-228]: *“Participants – who were from several European nationalities – acknowledged the cycling culture (e.g. using a bicycle for transportation) which is present in Dutch society.”*

42. Conclusion
    Page 25, line 578: How do the authors propose that researchers identify users and non-users of an intervention before an intervention is implemented?
    **Response:** This is indeed a very important question, thank you. We referred to this in our recommendations for future research. We feel that it would be very valuable if the non-users (and users) could be identified before an intervention is implemented. We adjusted the text accordingly [line 592-593*]: “Future research should look into how to identify users and non-users before the implementation phase and how to motivate them to adopt a practice period,…”*
