# Supplementary material for: The user and non-user perspective: Experiences of office workers with long-term access to sit-stand workstations
Source: PLoS One. 2020 Jul 28;15(7):e0236582. doi: 10.1371/journal.pone.0236582 (PMC7386596; doi:10.1371/journal.pone.0236582)
Supplement: Attachment — Submitted filename: Thanks again for the opportunity to re.docx [file pone.0236582.s005.docx]

Thanks again for the opportunity to re-review this paper. As I mentioned previously, I think this has a lot of potential. Although a lot of progress has been made to improving the standard of the paper, I would again suggest the authors resubmit a revised version. My main concerns are still in relation to the language used which was confusing and made the paper difficult to read and follow. I wouldn’t be confident it would be written clearly enough to be accessible to non-specialists. I found the language complicated and sentence structure clunky and difficult to navigate. Please consider reworking the grammar, syntax and choose less complicated terminology. Once this paper is more “reader friendly”, I think it will make a good addition to the literature. I do not feel this is presented in an intelligible fashion and written in standard English in some places. I am not clear on a number of items which make the interpretation of the results difficult. For example, I find the familiarisation and practise concepts difficult to understand without more information. A

Line 17: May benefit from insertion of country where the organisation is.

Line 18: From what I can see, open and axial coding is mentioned in this line and then not mentioned again in the manuscript. Please either further explain open and axial coding in the main text or consider removing from abstract.

Line 23: Is the term self-activation the best to use here? Please consider an alternative as it is not very clear what is meant. The term “activation” draws my mind to psychological theories.

Line 26: Consider clarification of practise periods and familiarisation here and throughout the paper. Will come back to this point when I meet it in the main body.

Line 28: Again will touch on this in main body, but more clarity around kinds of information is required here and main body.

Line 40: “INCLUDING SOLELY” please rephrase. Perhaps “only including”.

Line 40: I understand I mentioned this before and the advice was taken on board, but the English here isn’t the best. Perhaps use “device measured” in this instant to allow better flow. My apologies for confusion.

Line 42: 5.5 hours…. Per day? Please insert unit.

Line 46: If it has already been extensively evaluated, why is more research being done by you?

Line 46/47: Is this gain in popularity referring to the desks or the interest in occupational sitting levels etc.?

Line 48: Please be careful with terms here as inactive doesn’t mean sedentary, necessarily. Which is it? Or both?

Line 50: “INDEED”, is this a necessary word?

Line 57: Consider treadmill desk here.

Line 57: Is the word remarkable required here. In my opinion, here it doesn’t add to the scientific style of writing.

Line 57/58: Confusing sentence, unsure what is meant. Please rephrase for clarity.

Line 59/60: Please use the correct term “behaviour change wheel” and reference it (Michie).

Line 61: Again, correct term please. “Behaviour change techniques”. Please also reference.

Line 64/65: I don’t believe this is a solid rationale for why the qualitative methods were used. Please review.

Line 69-71: Please rewrite for clarity.

Line 89: Is the “at least in the past” at the end of this sentence needed, or is it repetition as mentioned earlier in the sentence?

Line 91-94: is this a recommendation for future research or a limitation of the study? Not sure how this fits here or the point being made. Please clarify.

Line 96-98: Please clarify here the link between the 2 studies more clearly. That one followed from the other, same research team, offices etc.

Line 99: Which study is being referred to?

Line 110: what is this referring to (±2000) plus or minus 2000? Employees? Please clarify in particular what the ± symbols is indicating here.

Line 112: Are all these desks then 21 years old? Are they dated? Or have the desks been upgraded?

Line 114: Please provide more precise information on the number of participants and the time of each session. Consider using descriptive stats for further clarity.

Line 124: Is this all that applied? How were they selected? Is this based on the inclusion/exclusion criteria?

Line 127-128: Please review the English used.

Line 130: Frequency of what?

Line 130-131: Why was collaboration required? Is it necessary to report this?

Line 134: What kind of consultation was available about the organisation?

Line 136: Why is gender an eligibility criteria?

Line 138: So you excluded some people based on their gender? Please explain

Line 140-142: Is this a requirement of the journal? I am not convinced this is critical to the paper if not required by journal specifications.

Line 147: Is this repetition?

Line 148-149: Unsure why this is relevant here.

Line 153: How was debate resolved?

Line 157: Why use word internal here? were there external too?

Line 167: Did you get much correction/comments, is this mentioned in results?

Line 176: I mentioned this earlier, here open coding only is referred to?

Line 182-183: Why was saturation not required, please include a reference for this.

Line 184-185: Was this only evident here?

Line 204: Please reconsider the use of the term attendant here.

Line 227-228: Why is the cycling culture relevant to occupational sitting?

Line 238: Is software a “facility”?

Line 244-248: Why so many quotation marks used here? Are they different quotes?

Line 253-256: This is an example of the hard to read sentences. Consider rephrasing and using randomly instead of random.

Line 256: How was this measured?

Line 317-318: How does the height impact conversation dynamic, please explain.

Line 335-337: I do not understand what is meant here.

Line 342-343: Please rephrase this sentence especially, “making them more prompted”.

Line 347: Is work place a better term here?

Line 365-367: hard to follow this sentence. Please reword.

Line 374: Please consider changing self-activation as mentioned previously.

Line 376: Does direction of switch matter here?

Line 386-387: Please consider only using the word strategy once in this sentence.

Line 391-392: As above, use/user is used 3 times. Please reword.

Line 393: Please consider using “in an upright position”, rather than “up”.

Line 394 (2^nd^ half): Is there a quote for increasing intervals?

Line 395: Is this an absence of use or physical absence from the office?

Line 402: What is the reason for the brackets here?

Line 406: Is promptious a typo? I can’t find it in a dictionary.

Line 406-407: Why so many quotation marks here also?

Line 410: What is a digital height indicator?

Line 411-412: Why so?

Line 419: provide information on (benefits of) using the standing option- is this relating to the desk use or just in general?

Line 419-420: What kind of information?

Line 420-421: Their role in doing what exactly?

Line 433: Is this double “or” required?

Line 433-435: Is this 2 points in the one sentence? The first half seems not to fit with the second half?

Line 436 “Active sitting”, what does this mean here? does the quote reflect this?

Line 444-446: please reword, what are the similar dialogues? from where?

Line 453-458: Could be made more concise? A lot of repetition here. What kind of threshold of acquaintance? is this the best term to use? i am not sure how a familiarisation period would work? Please explain what is meant here?

Line 471: “for users or non-users”, would and fit better here?

Line 472 and throughout: Is distinctive the best term? Consider something like "different"?

Line 479: “Practice period”, is this linking back to the acquaintance threshold etc.? Please ensure consistent terminology to avoid confusion.

Line 484-485: What does this sentence add? it might be worth summarising for readers the kinds of tasks if this adds to the paper.

Line 489-490: Was back not mentioned too?

Line 490: “They seemed hardly aware”- Please rephrase.

Line 496: How was the integration explored in this study? What does it mean?

Line 497: Please be more specific, little or none?

Line 502: Can you really say this definitively? Perhaps the word suggested is better here?

Line 507-509: please reword to avoid confusion.

Line 513: Does this mean both groups had the same knowledge/concerns about prolonged sitting?

Line 516: UK or US spelling?

Line 517: Is it a reason for not using it? Is reason the best word here? is it more of a side effect or something along those lines?

Line 526: Why the brackets?

Line 527-529: Throughout, what is meant by this practise period isn’t clear to me. Is it more like an introductory phase?

Line 530-531: How can you say this if they haven’t changed anything?

Line 533: “the persuasion by practice periods”- Unclear what is meant here.

Line 537: Our study? Or this study?

Line 543: Including/included x 2, please rephrase.

Line 546: please clarify why the placing of a desk result in psychological discomfort?

Line 551-552: Why is this identification important? do you just mean by asking someone if they think they’d use? Is this before they received a desk? Please elaborate.

Line 562-563: I do not grasp what is meant here. Please clarify.

Line 565: In the workplace population you looked at or all over the world?

Line 568: Did each person have their own individual office and their own individual desk? Did I miss this earlier? Apologies if I did. Please consider the impact this may have particularly on role modelling of others given that you cannot see the others in the building.

Line 572-573: Would benefit from a reference here.

Line 577-578: Do you think this happened? What was in place, if anything, to stop this?

Line 582-584: Please consider splitting into 2 sentences.

.
