# Supplementary material for: The user and non-user perspective: Experiences of office workers with long-term access to sit-stand workstations
Source: PLoS One. 2020 Jul 28;15(7):e0236582. doi: 10.1371/journal.pone.0236582 (PMC7386596; doi:10.1371/journal.pone.0236582)
Supplement: Attachment — Submitted filename: Response to reviewers.docx [file pone.0236582.s006.docx]

Response to reviewers

We would like to thank the editor and reviewers for their feedback and we appreciate the opportunity to resubmit this manuscript. We have addressed all comments from both reviewers point-by-point here below. The quoted texts from the manuscript are referred to with line numbers within the revised manuscript, however, the cited text is without track-changes to add clarity. Still, when needed the revised text is emphasised by underlining it.

Reviewer 1.

1. Thanks again for the opportunity to re-review this paper. As I mentioned previously, I think this has a lot of potential. Although a lot of progress has been made to improving the standard of the paper, I would again suggest the authors resubmit a revised version. My main concerns are still in relation to the language used which was confusing and made the paper difficult to read and follow. I wouldn’t be confident it would be written clearly enough to be accessible to non-specialists. I found the language complicated and sentence structure clunky and difficult to navigate. Please consider reworking the grammar, syntax and choose less complicated terminology. Once this paper is more “reader friendly”, I think it will make a good addition to the literature. I do not feel this is presented in an intelligible fashion and written in standard English in some places. I am not clear on a number of items which make the interpretation of the results difficult. For example, I find the familiarisation and practise concepts difficult to understand without more information.

**Response:** Thank you for your suggestions. We feel that they have further improved our manuscript. Please see our responses to the comments here below, in which we address each point made in detail.

2. Line 17: May benefit from insertion of country where the organisation is.
   **Response:** We have inserted “the Netherlands” as location of the organisation.
3. Line 18: From what I can see, open and axial coding is mentioned in this line and then not mentioned again in the manuscript. Please either further explain open and axial coding in the main text or consider removing from abstract.

**Response**: Thank you for pointing this out. During the previous revision, we have overlooked this. As we have now clarified our thematic analyses according to Braun and Clarke (2006), we have deleted the term axial coding from the abstract. Please also see our response to comment #14 of reviewer 2.

4. Line 23: Is the term self-activation the best to use here? Please consider an alternative as it is not very clear what is meant. The term “activation” draws my mind to psychological theories.
   **Response:** We have adjusted this and renamed the terms. In the results section it is mentioned and explained in detail, and in the abstract it reads as follows [line 23-24]:  *“Experiencing immediate benefits, including staying alert and increasing focus, were indicated by users as major facilitators for use”*

And in the result section we have specified this: [401-403]: *“This reason to use the standing option was specified by staying alert (a way to activate oneself when feeling drowsy) and increasing focus during work (when a drop in concentration was experienced).”*

5. Line 26: Consider clarification of practise periods and familiarisation here and throughout the paper. Will come back to this point when I meet it in the main body.
   **Response:** Thank you**,** in line with other comments regarding this terminology and as suggested by the reviewer in comment #88, we have adjusted this term into introductory phase to get familiar with standing. Here it now reads as follows [line 25-27]: *“Strategies to increase the use of the standing option included an introductory phase to become familiar with working while standing…”*
6. Line 28: Again will touch on this in main body, but more clarity around kinds of information is required here and main body.

**Response:** To enhance clarity, we have deleted providing information from the abstract and only included ‘providing reminders’. Furthermore, in the main body we have explained this according the following comments of the reviewer (#66, 67). The abstract now reads as follows [line 28-30]: *“Furthermore, providing reminders were mentioned as strategies to increase and sustain the use of the standing option.”*

7. Line 40: “INCLUDING SOLELY” please rephrase. Perhaps “only including”.

**Response:** We have rephrased this as suggested by the reviewer: [line: 41-42]: *“Furthermore, a recent meta-analysis, only including studies with* *device-measured sitting time,…”*

8. Line 40: I understand I mentioned this before and the advice was taken on board, but the English here isn’t the best. Perhaps use “device measured” in this instant to allow better flow. My apologies for confusion.
   **Response:** Thank you for clarifying this. We have adjusted it now, as shown in the text quoted in response to comment #7.
9. Line 42: 5.5 hours…. Per day? Please insert unit.

**Response:** We have inserted the unit “per day” here [line: 43-44]: “*In office workers, prolonged sitting is highly prevalent [9, 10], with almost 5.5 hours per day of sitting at the workplace…”*

10. Line 46: If it has already been extensively evaluated, why is more research being done by you?
    **Response:** Research to date has primarily focussed on quantitative evaluations of sit-stand workstations intervention and much less focus has been on long-term use of such workstations and participants’ experiences with these workstations, outside intervention efforts. We have now clarified this as follows [line: 46-48]: *“…interventions to reduce sedentary behaviour at the office, including sit-stand workstations, have been increasingly implemented and extensively evaluated as part of intervention programmes.”*

Furthermore, we have clarified the need for qualitative research, as indicated in the response to the reviewer’s comment #19.

11. Line 46/47: Is this gain in popularity referring to the desks or the interest in occupational sitting levels etc.?
    **Response:** The gain in popularity is referring to the sit-stand workstations, which are increasingly implemented. We have clarified this as follows [line 49-52]: *“…the gain in popularity of these interventions might be promising, since breaking up sitting time could reduce health risks associated with prolonged sitting in people who do not meet the physical activity guidelines of the World Health Organisation.”*
12. Line 48: Please be careful with terms here as inactive doesn’t mean sedentary, necessarily. Which is it? Or both?
    **Response:**  Thank you for pointing this out. Indeed, these terms are often used interchangeably, while they do not mean the same. Here we refer to inactive as the term is intended: not meeting the physical activity guidelines recommended by the WHO. For people who do not meet these guidelines, it has been shown that breaking up their sitting pattern might be beneficial for health risks associated with prolonged sitting. Please see our response on comment #11.
13. Line 50: “INDEED”, is this a necessary word?
    **Response:**  We have deleted this word and it now reads as follows [line: 54]: *“Recent studies on the use of sit-stand workstations found reductions in sitting time…”*
14. Line 57: Consider treadmill desk here.
    **Response:**  Thank you, this is indeed a better term. We have changed walking desk into treadmill desks, as suggested by the reviewer.
15. Line 57: Is the word remarkable required here. In my opinion, here it doesn’t add to the scientific style of writing.
    **Response:**  The term “remarkable” has been removed.
16. Line 57/58: Confusing sentence, unsure what is meant. Please rephrase for clarity.
    **Response:**  We have clarified this sentence by stating that it refers to strategies of individual office workers [line: 62-63]: *“Individual office workers seem to use many different strategies to reduce their sitting time and to replace it with standing or physical activity.”*
17. Line 59/60: Please use the correct term “behaviour change wheel” and reference it (Michie).
    **Response:**  Thank you for pointing this out. We have changed the term into behaviour change wheel and referenced it. The same applies to your next comment [line: 65-68]: *“In another study, the behaviour change wheel (Michie 2011) was used to develop a tailored intervention to reduce sitting, which identified 39 behaviour change techniques (Michie 2013) aimed at providing information, rewards, and prompts to reduce sitting time.”*
18. Line 61: Again, correct term please. “Behaviour change techniques”. Please also reference.
    **Response:**  Please see our response to the previous comment.
19. Line 64/65: I don’t believe this is a solid rationale for why the qualitative methods were used. Please review.
    **Response:**  We have now changed the phrasing to better clarify why qualitative research is needed [line 67-72]: *“To increase the reach of interventions, it is important to know why some office workers adopt the use of the standing option of their sit-stand workstations and why others do not and whether other strategies are being used to reduce sitting. To examine this, qualitative research, which aims to explore and better understand situations or behaviours, should be conducted [Pope and Mays, 1995].*
20. Line 69-71: Please rewrite for clarity.
    **Response:**  We have rewritten the sentence to increase clarity [line:76-78]: *“In these studies among sitting-based office workers it was found that workers could potentially be motivated to reduce their sitting time by providing them with information and raising their awareness about the health risks of prolonged sitting.”*
21. Line 89: Is the “at least in the past” at the end of this sentence needed, or is it repetition as mentioned earlier in the sentence?
    **Response:**  Thank you for pointing out this repetition. We have deleted this phrase from the sentence.
22. Line 91-94: is this a recommendation for future research or a limitation of the study? Not sure how this fits here or the point being made. Please clarify.
    **Response:** We were trying to make clear that a research gap exists. We have now clarified this as follows [line 101-104]: *“To our knowledge, the perspective of non-users of interventions such as sit-stand workstations, who have had access to the furniture at their workplace, without requesting it in the past, has not been included in previous studies. When the non-user profile is included, a profound understanding of both reasons for use and non-use of sit-stand workstations could be established.”*
23. Line 96-98: Please clarify here the link between the 2 studies more clearly. That one followed from the other, same research team, offices etc.
    **Response:**  We invited participants from the survey study to participate in this qualitative study, which was conducted by the same research team. We have clarified this as follows [line 107-110]: *“In a recent survey study about the use of sit-stand workstations within a large office population (N=1098) with long-term access to the furniture, we identified three user groups: daily users; monthly/weekly users; and non-users [36]. Users and non-users from this survey study, which was conducted by the same research team, were invited to participate in the present qualitative study.”*
24. Line 99: Which study is being referred to?
    **Response:**  We referred to our previous survey study in which we identified the user profiles within this office population (ref #36, Renaud et al. 2018). We have added this to the sentence, see our answer to the previous comment.
25. Line 110: what is this referring to (±2000) plus or minus 2000? Employees? Please clarify in particular what the ± symbols is indicating here.
    **Response:**  Thank you for asking, we have now clarified this as over 2000 employees [line 122]:  *“All employees (over 2000 in total) at this worksite…”*
26. Line 112: Are all these desks then 21 years old? Are they dated? Or have the desks been upgraded?
    **Response:**  Indeed this indicates that most desks were 21 years old. However, if desks were broken, they were replaced, and if new employees needed new desks, they were purchased. At the time of the study the workstations were all working properly.
27. Line 114: Please provide more precise information on the number of participants and the time of each session. Consider using descriptive stats for further clarity.

**Response:** In our results section we presented descriptives for all users, non-users and managers and internal ergo-coaches. Due to privacy reasons, we are unable to show these stats per group interview, as one of these group interviews only included two participants.

28. Line 124: Is this all that applied? How were they selected? Is this based on the inclusion/exclusion criteria?
    **Response:**  Thank you for pointing this out. The user profile was of course also one of the selection criteria. We have adjusted this section as follows [132-136]: *“Potential participants were eligible for inclusion if they: 1) had a user profile (self-reported average use of the sit-stand workstation of at least once per week to several times per day) or a non-user profile (self-reported average use of the sit-stand workstation of less than once per week), 2) indicated interest in participating in further research, and 3) provided their email address.”*
29. Line 127-128: Please review the English used.
    **Response:**  We have rewritten this sentence to improve the English language. Furthermore, because we have adjusted this section according to the recommendation made in the comment above, this now reads [line 139-142]: *“The invitees included 70 users, 47 non-users, and 27 managers.”*
30. Line 130: Frequency of what?
    **Response**: we have specified that this indicates the frequency of use of the sit-stand workstation.
31. Line 130-131: Why was collaboration required? Is it necessary to report this?
    **Response:**  We collaborated with the OHS department to be able to reach out to the internal ergo-coaches, since they knew who the internal ergo-coaches were. However, since this information is not necessary to understand the study, we have followed the suggestion by the reviewer and have deleted it from the sentence [line 145-147]: *“Furthermore, employees with a specific training for internal ergo-coach were all invited…”*
32. Line 134: What kind of consultation was available about the organisation?
    **Response:**  We have clarified this and now state that the consultations include the set-up of the workstations [line:146-148]: *“Internal ergo-coaches are employees who were trained in the ergonomic principles of a healthy workplace and could be consulted by their colleagues about the ergonomic set-up of their workstations.”*
33. Line 136: Why is gender an eligibility criteria?
    **Response:**  We see now that using the word “eligibility” may have been confusing. Gender was not an eligibility criteria, but served as characteristic of our population, we have clarified this as follows [line 150 -152]: *“If potential participants agreed to participate, they were asked to respond to the invitation email and answer some questions about eligibility (frequency of use, job profile, availability) and participant characteristics (gender and age).”*
34. Line 138: So you excluded some people based on their gender? Please explain
    **Response:** No participants were excluded based on their gender. We have clarified this in our manuscript as follows [line: 153-155]: *“In the selection of participants for the groups with specific user- and job profiles, we aimed to maximize variation in participant characteristics, such as gender and age, however we did not exclude any employee from participation.”*
35. Line 140-142: Is this a requirement of the journal? I am not convinced this is critical to the paper if not required by journal specifications.
    **Response:**  A description of the research team and the expertise of its members are a requirement for the COREQ checklist, hence we included this in the manuscript. However, as suggested by the reviewer we have now deleted this description of the expertise of the research team, to increase readability.
36. Line 147: Is this repetition?
    **Response:**  No this is not a repetition. This is referring to another (small) study, which we also conducted in this office population. [line 162-165]: *“Some of the participants, involved in the primary work process of the organisation, were familiar with the researcher (LR), because of participation in another, small (N=49) quantitative project…”*
37. Line 148-149: Unsure why this is relevant here.
    **Response:**  We have deleted the prior information about the research team (see response to the previous comment #35). This information about experience in occupational ergonomics is relevant as it indicates how interpretations of members of the research team potentially could be influenced by the work experience of the coders. According to QOREC it is requested to be open about these potential influences and to describe the expertise of the research team.

    Line 153: How was debate resolved?
    **Response:**  Debates were usually resolved with consensus about the strongest argument. We have provided a description about the content of the debate now [line: 168-171]: *“A researcher (LR) provided (sub-)questions based on (qualitative) literature to date, which were set in the definitive format after debate within the research team on relevance and suitability for the target population.”*
38. Line 157: Why use word internal here? were there external too?
    **Response:**  Yes, there were also external occupational health experts who could be consulted about the workstation set-up. We explained the concept of internal ergo-coach in the method section, and we try to use the term internal ergo-coach consistently. Please see our response to your comment #32 in which we explain the role of internal ergo-coaches.
39. Line 167: Did you get much correction/comments, is this mentioned in results?
    **Response:**  Thank you for pointing this out. We have explained now that the comments did not change the content of the summaries [line: 183-184]: *“Comments or corrections from participants on these summaries, which involved minor changes only, were processed.”*
40. Line 176: I mentioned this earlier, here open coding only is referred to?
    **Response:** In response to the comment of reviewer 2, we have changed this section in accordance to the article of Braun and Clarke (2006) about thematic analysis. Please see our response to comment #14 of reviewer 2.
41. Line 182-183: Why was saturation not required, please include a reference for this.
    **Response:**  Thank you for pointing this out. Saturation was not the explicit goal within this research. Because of the inclusion of many different perspectives we anticipated that more information was to be provided than when we would have only included one perspective, from the user-profile for example. Still, to reduce the potential confusion about this statement, we have rewritten this sentence as follows [line: 206-208]: *“Saturation seemed only to be established for one of the two identified major themes, since no new codes were identified during coding of the last parts of the final transcript.”*

Furthermore, in the limitation section of the discussion, we have now further elaborated on this [615 – 618]: *“Because of the variation of perspectives, more information was provided from different angles and it was anticipated that saturation would be difficult to be met for the two major themes. Still, reasons for use and non-use seemed saturated when the coding of all transcripts was finished.*”

42. Line 184-185: Was this only evident here?

**Response:**  Yes. Saturation was only evident for the major theme “reasons for use and non-use”.

43. Line 204: Please reconsider the use of the term attendant here.
    **Response:**  Thank you for pointing this out. We now have used the more commonly used term participants here.
44. Line 227-228: Why is the cycling culture relevant to occupational sitting?
    **Response:**  We have clarified why this is important by adding this [line: 250-251]: *“Participants – who were from several European nationalities – acknowledged the cycling culture (e.g. using a bicycle for transportation to and from work) present in Dutch society.”*
45. Line 238: Is software a “facility”?
    **Response:**  Thank you for pointing this out. We have changed this term so that it incorporates also the software: [line: 261-262]: *“Furthermore, occupational health support was offered to the employees, ranging from computer rest-break software to numerous sport facilities.*
46. Line 244-248: Why so many quotation marks used here? Are they different quotes?
    **Response:** It is a quote within a quote. To make this more clear we used single quotations now [line 268-269]: “….*but a colleague told me once ‘but that is when you do not pay attention that you are sitting crooked and that is when you run into trouble afterwards’.*
47. Line 253-256: This is an example of the hard to read sentences. Consider rephrasing and using randomly instead of random.
    **Response:**  Thank you for pointing this out. We have made two sentences of the text to increase clarity and added “randomly” in the description [line: 276-278]: *“Users reported several frequencies and durations of use of the standing option. Some indicated to use it with a clear pattern in switching from standing to sitting, while others used it randomly without a specific pattern.”*
48. Line 256: How was this measured?
    **Response:**  These were self-reported, as we have now indicated in the manuscript [line: 279-280]: *“Participants mentioned that durations of standing episodes lasted from 15 to 30 minutes up to several hours.”*
49. Line 317-318: How does the height impact conversation dynamic, please explain.
    **Response:** Thank you for pointing this out. We have now clarified this [line: 340-342]: *“Rationales behind putting the workstation up included to be at the same level as the visitor, shorter meetings and more intense conversations while being standing.”*
50. Line 335-337: I do not understand what is meant here.
    **Response:**  We have specified that, for non-users, experiencing discomfort due to the inability to find the correct standing position, was a reason for non-use [line: 358-362]: *“Experiencing discomfort from being in a standing position was also identified as a reason for non-use by non-users. However, this experience of discomfort was related to the inability of finding a correct standing position at the workstation rather than prolonged standing itself.”*
51. Line 342-343: Please rephrase this sentence especially, “making them more prompted”.
    **Response:**  We have now rephrased this sentence into [line 367-368]: *“From the user perspective, using the standing option was indicated as making it more easy to move around at the office and helped them to be physically active.*
52. Line 347: Is work place a better term here?
    **Response:**  Yes it is, thank you. We have changed work site into “workplace”.
53. Line 365-367: hard to follow this sentence. Please reword.
    **Response:** We have rewritten this sentence to increase readability [line 390-394]: *Experiencing or avoiding musculoskeletal complaints, such as low back pain, were indicated by users as reason to use the sit-stand workstation and by non-users as potential reason to start using the sit-stand workstation, respectively.”*
54. Line 374: Please consider changing self-activation as mentioned previously.
    **Response:**  We have followed the suggestion of the reviewer and we have changed this terminology. It now reads [line: 401-403]: *“This reason to use the standing option was specified by staying alert (a way to activate oneself when feeling drowsy) and increasing focus during work (when a drop in concentration was experienced).”*
55. Line 376: Does direction of switch matter here?
    **Response:**  Thank you for pointing this out, indeed it does. In this context, a switch from sitting to standing was meant, as we have now also clarified in the manuscript [line: 403-404]: *“This was explained as a switch to a standing posture, enabling a switch in focus during work.”*
56. Line 386-387: Please consider only using the word strategy once in this sentence.

**Response:**  We have addressed this as follows [line: 414-415]: *“The other theme included solutions “addressing other strategies”, which included subthemes aimed at organisational, office environmental, and individual adjustments.”*

57. Line 391-392: As above, use/user is used 3 times. Please reword.
    **Response:**  We have rewritten this particular sentence and the next to avoid using use/user that often in one sentence [line: 419-421]: “*To address the habitual reasons for non-use, it was stated that the use of the standing option should be incorporated within daily routines. To achieve that, several tips were provided by users, mostly aimed at a personal change in habits.”*
58. Line 393: Please consider using “in an upright position”, rather than “up”.
    **Response:**  As suggested, we have changed this into [line 421-422]: *“These included leaving the desk in an upright position when leaving the office and putting it in an upright position at fixed times.”*
59. Line 394 (2^nd^ half): Is there a quote for increasing intervals?
    **Response:**  We agree that a quote to indicate the importance of increasing intervals would be valuable, however, we also wanted to make clear that the risk of relapse also exists in users. Therefore, we have included the current quote [line: 425-428]: *“Every time you leave, raise the table. Because then you get the habit. And once you have the habit, then it is easy to follow because then you can just follow your body [...] The only problem I had was to restart after long holidays or after being really sick […] then you sit more at some days and then you have to consciously switch it on again.”*

Internal Ergo-coach

60. Line 395: Is this an absence of use or physical absence from the office?
    **Response:**  We have included absence from the office in this sentence to clarify this [line: 422-424]: “*Also, slowly increasing the intervals of use were recognised as a factor for successful incorporation, with prolonged periods of absence from the office increasing risks of relapse.*”
61. Line 402: What is the reason for the brackets here?
    **Response:**  We used brackets here to indicate that the routine might be new, but may also be already long-existing. However, we have decided to include both options and changed the sentence into [line: 430-431]: *“Reminders on a daily basis, for example from the rest-break software already available at personal computers, were mentioned to help to maintain new or existing routines.”*
62. Line 406: Is promptious a typo? I can’t find it in a dictionary.
    **Response:**  We tried to leave the quotes literally as was said by the participants. However, since this is indeed not an English word, we have changed the quote as follows [line: 436-437]: *“it can be a prompt seeing a colleague with their desk up and so I think ‘I have not done this for a while’.”*
63. Line 406-407: Why so many quotation marks here also?
    **Response:**  This is also a quote within a quote. We have added the single marks, in line with our response to comment #46.
64. Line 410: What is a digital height indicator?
    **Response:**  A digital height indicator shows the height of the sit-stand table in cm. In this way it is easy to see what the height of the desk is and in that way it is easier to set it to the desired sitting or standing height than without indicator. We have added the following text: [line 440-442]: *“…digital height indicators to show the height of the desks, preferably including a memory function to put the desk at a pre-set height, were suggested as solutions.”*
65. Line 411-412: Why so?
    **Response:**  We have deleted this sentence from the result section. In the discussion section we elaborate on why or why not practical solutions might increase the use [line 531-536]. *“Practical reasons for non-use, such as a desk surface which is full or too small (as only part of the workstations could be put up), were mentioned mainly by the non-users. Such practicalities have previously been mentioned as reasons for non-use in populations with short-term access to sit-stand workstations [31, 32]. However, when the use of the standing option is part of daily routines, this no longer seemed to be a practical barrier, as little of the users mentioned this.*
66. Line 419: provide information on (benefits of) using the standing option- is this relating to the desk use or just in general?
    **Response:**  This is related to the desk use, as we have now clarified this [line: 449-450]: *“…provide information about (health) benefits of using the standing option…”*
67. Line 419-420: What kind of information?
    **Response:**  This is referring to the desk use and (health) benefits of the use of the standing option. Please see our response to comment #66.
68. Line 420-421: Their role in doing what exactly?
    **Response:**  We meant their role in providing information. We have clarified this now as follows [line: 451-452] *“…the role that managers and ergo-coaches could play in providing this information was minimally acknowledged*.”
69. Line 433: Is this double “or” required?
    **Response:**  Thank you for pointing this out. We have deleted the word ‘or’ in this sentence.
70. Line 433-435: Is this 2 points in the one sentence? The first half seems not to fit with the second half?
    **Response:**  This was indeed not clear, but it was intended to fit both parts together. We have adjusted this and made two sentences, which now read as follows [line: 465-467]: *“It seemed important to adjust interventions to personal needs. It was suggested that a facility room could be set-up, where interested employees could try new tools such as desk bikes.”*
71. Line 436 “Active sitting”, what does this mean here? does the quote reflect this?
    **Response:**  This was also not clear enough, thank you for pointing this out. We have now adjusted it and describe the alternative for sitting, which includes active sitting solutions [line: 468-470]: *“Furthermore, it was emphasised again, by non-users, that sitting was comfortable to them and standing would not be an option, and sitting alternatives, such as active sitting chairs, were suggested.*
72. Line 444-446: please reword, what are the similar dialogues? from where?
    **Response:**  We have clarified this now [line 477-479]: *“Managers and ergo-coaches were predominantly users of the sit-stand workstations, which was reflected by similar reasons for use and non-use and similar strategies to increase standing and physical activity as mentioned by the users.”*
73. Line 453-458: Could be made more concise? A lot of repetition here. What kind of threshold of acquaintance? is this the best term to use? i am not sure how a familiarisation period would work? Please explain what is meant here?
    **Response:**  We have rewritten these sentences to explain more clearly what the ergo-coaches meant with the proposed practice period. Furthermore, we have changed the terminology according to the reviewer’s comment #88. Please see line 488-494]: *“They emphasised the importance of an introductory phase for potential users to familiarise themselves with standing. They indicated that this phase should last at least several days. After this, immediate benefits of using the standing option could be experienced and the use could be incorporated in daily routines. If the threshold of several days was not met, routine usage would fail, as was also revealed in a personal example.”*
74. Line 471: “for users or non-users”, would and fit better here?
    **Response:**  Thank you for pointing this out, this needs to be ‘and’ indeed; we have adjusted this accordingly.
75. Line 472 and throughout: Is distinctive the best term? Consider something like "different"?
    **Response:** Opposite to shared reasons for use and non-use, we feel that the term distinct, which was confused with distinctive, fits best here. We have altered this term into distinct throughout the manuscript now.
76. Line 479: “Practice period”, is this linking back to the acquaintance threshold etc.? Please ensure consistent terminology to avoid confusion.
    **Response:** Also, in response to the reviewer’s comment #73 and #88, we have used the terminology of introductory phase instead of practice period consistently throughout the manuscript now.
77. Line 484-485: What does this sentence add? it might be worth summarising for readers the kinds of tasks if this adds to the paper.
    **Response:**  We felt that the meetings while standing were an example of a work tasks which users indicated as a reason to put the workstation in standing height. However, we agree with the reviewer that there are more concise examples. We have followed the suggestion of the reviewer and have deleted this sentence and have included a more suitable example [line 519-523]: *“Users identified specific work tasks for which no high levels of concentration were required as a reason to put the workstation at standing height, for example going through emails. Specific work tasks, associated with standing have also been indicated in other studies.”*
78. Line 489-490: Was back not mentioned too?
    **Response:**  The back was predominantly mentioned as a reason to start using the standing option in daily routines. This particular sentence dealt with reasons for returning to the sitting position after a single period of standing and the back was not mentioned here.
79. Line 490: “They seemed hardly aware”- Please rephrase.
    **Response:**  Thank you, we have rephrased this into [line: 528-529]: *“They seemed unaware of the risks of prolonged standing such as varicose veins…”*
80. Line 496: How was the integration explored in this study? What does it mean?
    **Response:**  We have rewritten this sentence and it now reads [line: 534-536]: *“However, when the use of the standing option is part of daily routines, this no longer seemed to be a practical barrier, as only few users mentioned this.”*
81. Line 497: Please be more specific, little or none?
    **Response:**  Please see our response to the previous comment.
82. Line 502: Can you really say this definitively? Perhaps the word suggested is better here?
    **Response:**  Indeed we cannot say with complete certainty that the work productivity is not affected by the use of sit-stand workstations. However, the evidence is quite extensive, as indicated by the reviews we refer to, all showing that the work productivity is not affected. Still, we do not state it too definitely, indicated by the word ‘might’. The word ‘suggested’ is in our view not strong enough here. [540-542]: *“Although it has been shown in quantitative research that work productivity might not be affected when sit-stand workstations are used [21, 42, 43]…”*
83. Line 507-509: please reword to avoid confusion.
    **Response:**  We have rewritten this section as follows to clarify [line 546-549]: *“In other studies this has been indicated as high focus work tasks being prioritised over the use of the standing option [39], or as the sitting posture which is not consciously experienced during high-focus work tasks [25].”*
84. Line 513: Does this mean both groups had the same knowledge/concerns about prolonged sitting?
    **Response:**  Yes indeed. Both users and non-users were informed about the health risks of prolonged sitting.
85. Line 516: UK or US spelling?
    **Response:**  We use UK spelling, we have changed it into recognised. Please see our quote in answer to the next comment.
86. Line 517: Is it a reason for not using it? Is reason the best word here? is it more of a side effect or something along those lines?
    **Response:** Thank you for pointing this out. We have rephrased this into [line: 556-558]: *“In line with a broadly recognised sitting culture, both users and non-users mentioned that the persistent habit of sitting withheld them from using the standing option (more often).”*
87. Line 526: Why the brackets?
    **Response:** We have deleted the brackets now, this was indeed not necessary.
88. Line 527-529: Throughout, what is meant by this practise period isn’t clear to me. Is it more like an introductory phase?
    **Response:**  Thank you for pointing this out, this indeed what we mean by this and we have now adjusted the term into introductory phase throughout the paper. Here it reads as follows [line: 565-569]: *“When developing interventions including sit-stand workstations (environmental restructuring), adopting an introductory phase (persuasion) might lead to immediate experienced benefits, as indicated by the users in our study, which could result in long-term use.”*
89. Line 530-531: How can you say this if they haven’t changed anything?
    **Response:**  Thank you for pointing this out. We could have been more clear that this was indicated by the users, as they have had experience with this. We have added this information to this sentence [line: 569-571]: *“However, it was also indicated by the users that this introductory phase takes time and energy…”*
90. Line 533: “the persuasion by practice periods”- Unclear what is meant here.
    **Response:**  In line with our response to the reviewers previous comment (#88) this has been adjusted as follows [line: 575- 576]: *“Still, the introductory phase to induce long-term use, might also be needed in such ‘non-user’ interventions.”*
91. Line 537: Our study? Or this study?
    **Response:**  We have adjusted this according to the suggestion of the reviewer into “this study”.
92. Line 543: Including/included x 2, please rephrase.
    **Response:**  Thank you for pointing this out. We have adjusted this as follows [line: 585- 586]: *“Standing (group) meetings, including the proper furniture, might be implemented as a strategy…”*
93. Line 546: please clarify why the placing of a desk result in psychological discomfort?
    **Response:**  We referenced a study which has shown that participants experienced physiological and psychological discomfort during a standing meeting using standing tables. We have now specified this in our manuscript as follows [line: 587-589]: *“Still, there might be more needed than just placing standing tables in a meeting room to overcome the sitting culture, as the use of these tables might also introduce physical and psychological discomfort during meetings.”*
94. Line 551-552: Why is this identification important? do you just mean by asking someone if they think they’d use? Is this before they received a desk? Please elaborate.
    **Response:** We suggested to further investigate whether it could increase the reach of interventions if identification of the user profile beforehand would be possible. We have now clarified this as follows [line: 594 – 596]: *“Future research should look into how to identify employees who are potential users or non-users and whether differentiating between them could increase the reach and effectiveness of interventions.”*
95. Line 562-563: I do not grasp what is meant here. Please clarify.
    **Response:**  With this sentence we wanted to explain what the potential selection bias may have resulted into, namely the possible selection of the more enthusiastic and physically active non-users in our study [line 606-608]: *“Therefore, the non-users we included in our study might have been more enthusiastic and physically active, which may have resulted in similar contextual and individual factors between users and non-users.”*
96. Line 565: In the workplace population you looked at or all over the world?
    **Response:** We have clarified now that this involves the population of the study [line 608-609]: *“In the total study population …”*
97. Line 568: Did each person have their own individual office and their own individual desk? Did I miss this earlier? Apologies if I did. Please consider the impact this may have particularly on role modelling of others given that you cannot see the others in the building.
    **Response:**  Yes, most employees have a personal office. However, they have an open door policy, making it possible to see each other when walking through the hallways. Furthermore, seeing each other was suggested by the participants themselves as a possible reminder to use the standing option. Still, a personal office is not very common these days. Hence, this was indicated as a limitation of our study regarding the generalisability towards other office populations.
98. Line 572-573: Would benefit from a reference here.
    **Response:**  In response to comment (#41) we clarified that saturation was met for one major theme. Please see our response, in which we also address the changes made in the limitation section of the discussion.
99. Line 577-578: Do you think this happened? What was in place, if anything, to stop this?
    **Response:**  We indeed felt that this influence could not to be stopped, therefore, we mentioned it as a limitation of the study. However, we consider this potential influence limited, which we have added to the discussion [line 619-624]: *“Because of the clear research aim (to gain insight in reasons for use and non-use of sit-stand workstations as well as insight in other strategies to decrease sitting and increase physical activity in the workplace), the coding process might have been influenced beforehand.* *However, all themes within the two major themes, and the extra separate theme, resulted from the data analysis process, suggesting that the influence was limited.*”
100. Line 582-584: Please consider splitting into 2 sentences.
     **Response:**  Thank you for this suggestion, we have split this sentence in two parts. It now reads as follows [line 627-629]: *“Users and non-users of sit-stand workstations identified shared and distinct reasons for use and non-use of the standing option. Furthermore, they provided insight in potential strategies to increase standing and physical activities at the workplace.”*

Reviewer #2:

The authors have attended to my comments well and I believe it has strengthened the paper greatly. I do agree with Reviewer One, who stated in their review that in places the manuscript was difficult to follow. While I believe this has been addressed in places, I have made a number of comments below indicating where there are some grammatical/ spelling/ formatting errors that when corrected will further improve the flow of the manuscript.

The Line numbers I have used refer to the document with tracked changes.

1. Throughout the manuscript, both UK and US English spelling are used. I would suggest referring to the journal guidelines about which form should be used, and amending throughout. For example line 516 recognized (US spelling) and line 523 organisational (UK spelling).
   **Response**: Thank you for pointing this out. We have used UK spelling now throughout the manuscript, and adjusted recognized into recognised accordingly.
2. Line 51- I suggest rewording this sentence to “could reduce health risks associated with prolonged sitting in people who are inactive”.
   **Response**: Thank you for pointing this out, we have adjusted this sentence now, as suggested by the reviewer [line: 50-51]: *“…could reduce health risks associated with prolonged sitting in people who do not meet the physical activity guidelines of the World Health Organisation.”*
3. Line 67- I am unclear from the description what the Stand Up Australia study actually entailed. I think further description is needed here (perhaps just a sentence about the study design/ methods).
   **Response**: We have added the purpose of the stand-up Victoria Study now. Although we have only added a few words, we feel it is now better explained [line 56-60]: *“Randomised controlled trials, conducted in large office populations, introducing multi-component interventions (combining several components, for example coaching, reminders and sit-stand workstations) and evaluating their effect, such as the Stand-Up Victoria study, showed similar reductions of around 45 minutes occupational sitting time per day at 12 months of follow-up [19, 20].”*
4. Line 76- qualitative used twice- sentence needs to be rephrased.
   **Response**: Thank you for pointing this out, we have deleted the word qualitative the second time. The sentence now reads [line 74]: *“A synthesis of qualitative research [27] included studies among sitting-based office…”*
5. Line 82- check grammar here
   **Response**: We have changed this sentence in order to correct the grammar as follows [line: 81-83]: *“The most important motivation to reduce sitting time seemed to be enhancing musculoskeletal health and not reducing long-term health risks, such as diabetes or cardiovascular disease.”*
6. Line 98- typo “dependent of”
   **Response**: Thank you for pointing this out. We have change it into dependent on.
7. Line 117- should this read ergo-coaches?
   **Response**: Thank you, this has been adjusted into plural as suggested by the reviewer.
8. Line 127- consider changing the word obtained to installed.
   **Response**: We have changed this word according to the suggestion of the reviewer. This now reads as follows [line:123-124]: *“…access to electrically adjustable sit-stand workstations since the start of their employment or since 1999, when the furniture was installed.”*
9. Line 153- Does this mean that some individuals volunteered and were eligible to participate but were not selected? Does this have any implications in terms of bias this may have introduced?
   **Response**: We did not exclude any employee from participation based on their characteristics. We only used this information to be able to maximize the variation within the groups. We have explained this in the manuscript as follows, also in accordance with comment #34 of reviewers 1 [line: 153-155]: *“In the selection of participants for the groups with specific user- and job profiles, we aimed to maximize variation in participant characteristics, such as gender and age, however we did not exclude any employee from participation.”*
10. Line 171- if the phrase “based on literature to date” is to be used here, I would suggest a citation (or citations) to the pertinent literature is required here.
    **Response**: We have added the references to the manuscript now (Grunseit et al. 2013, Chau et al. 2014, DeCocker 2015) [line 168-169]: “*A researcher (LR) provided (sub-)questions based on (qualitative) literature to date [29, 31, 32],..”*
11. Line 180- check grammar here “get familiar with the topic”.
    **Response:** Thank you for pointing this out, we have adjusted this into [line 179-180]: *“This warm-up exercise for participants was intended to become familiar with the topic and the group*
12. Line 185- what was involved in the “processing of comments and queries” were any changes made to the interview transcripts?
    **Response:** We have clarified this in the manuscript, also in line with comment #39 of reviewer 1 [line: 183-184]: *“Comments or corrections from participants on these summaries, which involved minor changes only, were processed.”*
13. Line 192- check grammar “was done”
    **Response:** We have changed this, also in line with the reviewers next comment into [line 191-192]: *“…open coding was also conducted by MH and open coding of the management interview transcript was conducted by both ES and LR.”*
14. Line 188- While I commend the authors for including more detail in the data analysis section, I found it difficult to follow in parts. I am still unclear where each of the six steps for thematic analysis begin and end. Perhaps it would be useful to use phrases like “in the first/ second/ third stage of thematic analysis....” to guide the reader through this section.
    **Response:** Thank you for this suggestion. We agree that adding the actual six phases of thematic analysis provides more clarity. This paragraph now reads as follows [line:186-208]: *“Data were analysed using ATLAS.ti 8 software and included three data coders (LR, MH and ES). After data collection, the six steps of thematic (content) analysis were used [38] to identify themes, which involved open coding, and identifying, reviewing and defining (major) themes. Phase 1 included reading of the transcripts to become familiar with the data. Phase 2 included open coding of the four user and non-user interview transcripts, which was done by LR, to generate initial codes. For one interview transcript (non-user profile, supportive personnel), open coding was also conducted by MH and open coding of the management interview transcript was both conducted by ES and LR. During phase 3, which encompassed searching for themes, general codes between LR and both coders (MH and ES) were discussed. When differences occurred, consensus between the coders was sought. Additional codes from the coding of the transcripts by LR, were discussed with MH and consensus was sought again between coders. In phase 4 and 5, which encompassed reviewing and naming themes, the resulting initial coding tree was discussed between LR and HRP. Within the coding tree, two major themes and one extra separate theme with subthemes and main codes were reviewed and identified. This was again discussed within the whole research team and resulted in two final thematic maps, which encompassed the hierarchy of the two major themes. Furthermore, the hierarchy of the extra separate theme was set in a thematic map. Phase six included reporting on the data in the results section of this manuscript. Saturation seemed only to be established for one of the two identified major themes, since no new codes were identified during coding of the last parts of the final transcript.”*
15. Line 203: This sentence is quite unclear. I am unclear how a hierarchy of two major themes and an extra theme were presented in three conceptual frameworks. Is there a simpler and more concise way that this could be explained? Perhaps the authors could consider refer back to the language used by Braun and Clarke to guide the writing of this section?
    **Response:** We have altered the terminology in accordance with the previous comment of the reviewer. We now use the terminology used by Bran &Clarke. We have renamed the frameworks into the two major final thematic maps, and we have indicated that the extra theme was also graphically put into a thematic map. Please see the quoted text from the manuscript here above.
16. Line 332- typo “indicated as reason”
    **Response:** Thank you for noticing. We have adjusted this sentence as follows [line 329-330]: *“Performing work tasks for which a high level of concentration was needed, was mentioned as a reason for non-use, predominantly by users.”*
17. Line 496- consider changing “looked into” to “explored”
    **Response:** Thank you for pointing this out. We have changed this accordingly [line 502]: *“This study explored reasons for use and non-use of sit-stand workstations…”*
18. Line 499- consider rephrasing “the most important finding seemed that”
    **Response:** We have adjusted this as follows [line 505-508]: *“The most important finding was that users and non-user of the standing option indicated several similar reasons for use and non-use…”*
19. Line 525- consider rephrasing “switch back” to “return”. Switch back is colloquial language.
    **Response:** As suggested by the reviewer, we have changed this phrasing into [line 527-528]: *“…the experience of bodily discomfort in the lower extremities, was a reason to return to a seated position.”*
20. Line 532- I think the phrase “none to little” needs to be changed here. I think the authors need to specifically state whether it was a few participants that mentioned this, or none at all, as it cannot be both.
    **Response:** Indeed this phrasing was unclear, we have now changed this into [line: 535-538]: *“…this no longer seemed to be a practical barrier, as only few of the users mentioned this.”*
21. Line 592- should this phrase be “physically active” rather than “physical active”?
    **Response:** Yes indeed, thank you. We have adjusted it [line 592-593]: *“Interventions to reduce sitting at the office should include shared components, such as incorporating a more physically active work culture..*.”
22. Line 633- consider rephrasing “get familiarised” to “become familiarised”?
    **Response:** We have adjusted this phrasing into [line 635-636]: *“…in order to experience immediate benefits and become familiar with a new routine.*
