# Supplementary material for: The user and non-user perspective: Experiences of office workers with long-term access to sit-stand workstations
Source: PLoS One. 2020 Jul 28;15(7):e0236582. doi: 10.1371/journal.pone.0236582 (PMC7386596; doi:10.1371/journal.pone.0236582)
Supplement: Attachment — Submitted filename: This paper is now very much improved.docx [file pone.0236582.s007.docx]

This paper is now very much improved. Thanks for taking the time to revise.Below are some points to be addressed before publication.

Line 18: Is this the correct order of steps?

Line 28: Reminders to do what?

Line 29: What are you referring to when you say “this”?

Line 32:Look into—change to explore?

Line 33- is perceive best? Experience? Yet again, not all will experience the benefits, so need to find a word here to match both.

Line 47-50 , hard to read and unsure of point being made.

Line 58- consider change seemed to appeared ?

Line 72-74- please consider using a comma or commas to make the sentence more readable.

Line 75- alternative word for seemed

Line 89-90: How is peer support a personal consideration?

Line 90- Please explain what you mean by novelty here- it is not the same “novelty” you refer to about the novelty of the desks in the paragraphs above and this may confuse readers

Line 99-102 it is still not really clear here whether you conducted this survey study too? Is it a precursor study to the one being reviewed here? You identified the sub groups from your own previous work? Or from someone else’s work? Although it is mentioned by the authors, I think the order that things are mentioned could be clearer.

Line 163-164: English here could be tightened up.

Line 169- what implications did the processing have? Were changes to the topic guide etc. made based on comments?

Line 174-178: Why one coder for some, 2 for others?

Line 181-185: Unclear please re write. Data analysis section is still hard to follow in places especially 181-185

Line 186-188; unclear of the point being made re saturation. So it was or wasn’t reached? For some themes only? I am unfamiliar with saturation being reported in this manner, What implications does this saturation/lack of saturation have in the discussion and interpretation of results?

Line 230- not sure if oyu can be from a nationality? Perhaps from a country is more correct.

Line 230-231how does biking to work impact of sitting at work?

Line 322: Consider “while standing”

Line 424: Consider using the term “role model”, but then be careful of the frequency of the term “role” in this section.

Line 557-558: How cold you do this?

Line 559- what does shared components mean
