# Supplementary material for: The user and non-user perspective: Experiences of office workers with long-term access to sit-stand workstations
Source: PLoS One. 2020 Jul 28;15(7):e0236582. doi: 10.1371/journal.pone.0236582 (PMC7386596; doi:10.1371/journal.pone.0236582)
Supplement: Attachment — Submitted filename: Response to the reviewers.docx [file pone.0236582.s008.docx]

**Response to reviewers**

We would like to thank the editor and reviewers for their feedback and we appreciate the opportunity to resubmit this manuscript again. We have addressed all comments from both reviewers point-by-point here below and feel it has increased readability of the manuscript. We have uploaded the revised manuscript with track-changes and refer in our responses to the line numbers where the changes were made. In this response letter we have not used track-changes, but have underlined the text to emphasise changes.

1. Reviewer #1: This paper is now very much improved. Thanks for taking the time to revise. Below are some points to be addressed before publication.
   **Response:** Thank you, we have addressed each point here below.
2. Line 18: Is this the correct order of steps?
   **Response:** Thank you for pointing this out. It is the right order (as thematic analysis is referring to the overall methods used), but the steps taken may not have been completely clear. We have now adjusted this as follows [line 17-19]: *“The verbatim transcripts were analysed using thematic analysis. Open coding was conducted by three researchers and codes and themes were discussed within the research team.”*
3. Line 28: Reminders to do what?
   **Response:** We have now clarified that the reminders refer to the use of the standing option: [line 28-30]: *“Furthermore, providing reminders to use the standing option were mentioned as a strategy to increase and sustain the use of the sit-stand workstations.”*
4. Line 29: What are you referring to when you say “this”?
   **Response:** We were referring to the increased use of the sit-stand workstations, which we have now added to the manuscript [line 30-31]: *“Increased use may lead to a change in the sitting culture within the organisation, as more employees would adopt active movement behaviours.”*
5. Line 32: Look into—change to explore?
   **Response:** Thank you for this suggestion, we have altered the wording accordingly [line 33-35]: *“Future research should explore how to motivate potential users and non-users to adhere to an introductory phase in order to experience these immediate benefits,…”*
6. Line 33- is perceive best? Experience? Yet again, not all will experience the benefits, so need to find a word here to match both.
   **Response:** Thank you for this suggestion, we have adjusted this to experience, although indeed not all will experience the benefits. That is why we also have added that it concerns potential users and non-users. Please see our response to comment #5.
7. Line 47-50 , hard to read and unsure of point being made.
   **Response:** Thank you for pointing this out. We have changed the last part of the sentence in which we now have referenced the review of Prince et al (2019). We feel this sentence is more in line with the argument made in the paragraph above it now [line 48-53]: *“From a public health perspective, the gain in popularity of these interventions might be promising,* *because of their high potential to reduce sitting among office workers, who have shown to be overall highly sedentary [17].”*
8. Line 58- consider change seemed to appeared ?
   **Response:** Thank you for this suggestion, we have adjusted this in line with the reviewers’ suggestion [line: 61-62]: *“Furthermore, sit-stand workstations appeared not to affect work performance*.”
9. Line 72-74- please consider using a comma or commas to make the sentence more readable.
   **Response:** We have included a comma to make the sentence more readable [line: 75-77]: *“In these studies among sitting-based office workers it was found that workers could potentially be motivated to reduce their sitting time, by providing them with information and raising their awareness about the health risks of prolonged sitting.”*
10. Line 75- alternative word for seemed
    **Response:** We have changed this word as follows [line: 77-79]: *“The most important motivation to reduce sitting time according to these workers was to enhance musculoskeletal health…”*
11. Line 89-90: How is peer support a personal consideration?
    **Response:** This is indeed not the correct example. We have now added another result from this cited paper, which is better reflecting the personal considerations mentioned [line: 93-94]: *“…sustained use of the furniture was dependent on personal considerations such as the selection of a specific work task while using the standing option…”*
12. Line 90- Please explain what you mean by novelty here- it is not the same “novelty” you refer to about the novelty of the desks in the paragraphs above and this may confuse readers
    **Response:** Thank you for pointing this out, these are indeed two different concepts. We have now adjusted this sentence to add clarity [line: 94-95]: *“Although the inclusion of previous users added a new perspective, the previous users…”*
13. Line 99-102 it is still not really clear here whether you conducted this survey study too? Is it a precursor study to the one being reviewed here? You identified the sub groups from your own previous work? Or from someone else’s work? Although it is mentioned by the authors, I think the order that things are mentioned could be clearer.
    **Response:** We have adjusted the first part of the paragraph to better explain that we conducted this survey study and explain that we indeed, identified the users and non-users within this survey study [line: 104-108]: *“We have previously conducted a survey study about the use of sit-stand workstations within a large office population (N=1098) with long-term access to the furniture. In this study we identified three user groups: daily users; monthly/weekly users; and non-users [36]. Users and non-users from this survey study, were invited to participate in the present qualitative study.*”
14. Line 163-164: English here could be tightened up.
    **Response:** Thank you for pointing this out. We have changed this sentence into [line: 166-168]: *“Every group interview started with a warm-up exercise, in which couples of participants discussed and noted down reasons for use and reasons for non-use of the sit-stand workstations.”*
15. Line 169- what implications did the processing have? Were changes to the topic guide etc. made based on comments?
    **Response:**  Also in response to comment #9 of reviewer 2, we have clarified that this processing did not change the content of the summaries [line:173-175]: *“Comments or corrections from participants on these summaries, which involved minor changes only, were processed and did not change the content of the summaries.”*

Furthermore, the summaries were only used by the researchers to become familiar with the data, which was part of step one. We have now also specifically included this in the data analysis section [line: 178-180]: *“Phase one included reading of the summaries and the total transcripts to become familiar with the data.”*

16. Line 174-178: Why one coder for some, 2 for others?
    **Response:** The coding of the first transcripts was done by two coders. Because there appeared to be many similarities between the transcripts, the further coding process was done by one coder. To ensure triangulation of coders, all codes were also discussed by LR and MH and with HRP, as stated in the method section (data analysis paragraph).
17. Line 181-185: Unclear please re write. Data analysis section is still hard to follow in places especially 181-185
    **Response:** Thank you for pointing this out. We have clarified this now, also in line with comment #12 of reviewer 2. [line: 189-197]: *“Within the coding tree one extra separate theme “contextual and individual factors” with subthemes and main codes was identified. Additionally, two major themes, including themes, subthemes and main codes were reviewed and identified: 1) reasons for use and non-use of the standing option and 2) strategies to increase the use of the standing option and physical activity at the workplace. This was again discussed within the whole research team and the hierarchy of the extra separate theme and the two major themes were set in thematic maps.”*
18. Line 186-188; unclear of the point being made re saturation. So it was or wasn’t reached? For some themes only? I am unfamiliar with saturation being reported in this manner, What implications does this saturation/lack of saturation have in the discussion and interpretation of results?
    **Response:** In line with comment #8 of reviewer 2 we have elaborated about this in the discussion section. Please see our response to comment #8 of reviewer 2.
19. Line 230- not sure if oyu can be from a nationality? Perhaps from a country is more correct.
    **Response:** Thank you for pointing this out. We have changed this into [line: 242]: *“Participants – who were from several European countries …”*
20. Line 230-231how does biking to work impact of sitting at work?
    **Response:** This section describes themes/codes that were mentioned by the participants that were related to the cultural context. This cultural context included ways of behaviour that were linked to the Dutch culture, e.g. riding bikes is commonly used for transportation in The Netherlands. Participants brought these issues up during the group interviews. It is not stated here that this would specifically impact sitting at work, however, it was discussed by the participants, that is why we have presented it as an extra separate theme, describing the cultural context in which the participants live and work.
21. Line 322: Consider “while standing”
    **Response:** We have adjusted this sentence in line with comment #13 of reviewer 2. We have shortened the sentence and have deleted the part describing the conversations while standing. This sentence now reads [line 333-335]: *“Rationales behind putting the workstation up included to be at the same level as the visitor and shortening the meeting.”*
22. Line 424: Consider using the term “role model”, but then be careful of the frequency of the term “role” in this section.
    **Response:** Thank you for this suggestion, we have included the term role model now in this section [ line: 436-439]: *“Within organisational adjustments, managers and internal ergo-coaches acknowledged that they could be a role model for employees, by being an example and provide information about the (health) benefits of using the standing option and being less sedentary at the office.”*
23. Line 557-558: How cold you do this?
    **Response:**  We have provided an example of how to address health risks associated with prolonged standing, referring to the role models t[line: 572-574]: *“Furthermore, when implementing interventions to reduce sitting time, information about health risks associated with prolonged standing should also be provided [54, 55], for example by managers and internal ergo-coaches acting as role models.”*
24. Line 559- what does shared components mean
    **Response:** We have changed this term to make clear what we mean [line 575-576]: *“Interventions to reduce sitting at the office should include components that address all employees, such as incorporating a more physically active work culture…”*

Reviewer #2

1. Thank you for attending to my comments. I have made a number of further comments that I think will strengthen your manuscript. The line numbers refer to the manuscript with tracked changes.
   **Response:**  Thank you, we have addressed the comments of the reviewer here below.
2. Abstract:
   Line 17: Organization- UK or US spelling?
   **Response:** Thank you for pointing this out, we have changed it into UK spelling: organisation
3. Line 20: thematic coding or thematic analysis? Please ensure terminology is consistent throughout the manuscript.
   **Response:** Thank you for pointing this out, we have now used thematic analysis consistently throughout the manuscript.
4. Line 24: the authors state that “staying alert and increasing focus” are facilitators for the use of standing desks. Would these factors not be “perceived benefits” rather than facilitators? In my mind a facilitator would be something like workplace culture or management support (i.e. something that makes it easier for an individual to participate in the behaviour), rather than a reason/motivation/ perceived benefit of the activity. If this is to be changed here, it will also need to be changed throughout the manuscript.
   **Response:** Thank you for this suggestion. We agree with the reviewer that facilitator might not have been the most appropriate term here. However, overall we already describe this reason for use as “experienced benefits”. We have now reworded “major facilitator” into “important reason for use” [line 23-24]: *“Experiencing immediate benefits, including staying alert and increasing focus, were indicated by users as the most important reasons for use.”*

   Introduction
5. Line 54- suggest changing this to “Recent studies exploring the use of”.
   **Response:** We have adjusted this sentence in line with the suggestion of the reviewer [line 54]: *“Recent studies exploring the use of sit-stand workstations…”*
6. Line 56: Sentence starting “randomised controlled trials”: I realise the additions to this sentence have been due to a number of reviewer comments, however I think this sentence needs to be sense checked and perhaps split into two sentences. It is very long and difficult to read.
   **Response:** This sentence became indeed quite long during the reviewing process. We have now adjusted it as follows [line: 56-61]: *“Randomised controlled trials, such as the Stand-Up Victoria study, have been conducted in large office populations, introducing multi-component interventions (combining several components, for example coaching, reminders and sit-stand workstations). These evaluations showed similar reductions of around 45 minutes occupational sitting time per day at 12 months of follow-up [19, 20].”*
7. Line 76-83: Could these two sentences be condensed into one sentence to avoid repetition?
   **Response:** We have condensed the two sentences into one, as suggested by the reviewer [line: 80-85]: *“The synthesis also included qualitative studies conducted shortly after implementation of sit-stand workstations and, focussing on user-experiences of the furniture [31-33], identified support from co-workers and managers as an overall facilitator and a predominant sitting culture as an overall barrier to reduce occupational sitting time [27].*

Method

8. Line 182- perhaps this reference will assist the authors in their discussion around data saturation Clarke, V., & Braun, V. (2020). To saturate or not to saturate? Questioning data saturation as a useful concept for thematic analysis and sample-size rationales. Qualitative Research in Sport, Exercise and Health.
   **Response:** Thank you for this valuable reference. We have added this reference in the discussion section now, to support our statements around the proposed saturation. Furthermore, we have adjusted the phrasing in the method section as follows [line: 198-200]: *“Saturation seemed only to be established for one of the two identified major themes, since no new codes that considerably altered the coding tree, were identified during coding of the last parts of the final transcript.”*

Discussion section [line: 598-603]: *“Because of the variation of perspectives, more information was provided from different angles and it was anticipated that saturation would be difficult to be met for the two major themes. Still, reasons for use and non-use seemed saturated when the coding of all transcripts was finished. This major theme was elaborately discussed from all perspectives, and no new codes that considerably altered the coding tree seemed to come up, although this was subject to the interpretation of the researchers [Braun &Clarke 2019].”*

9. Line: 183-184: In my previous comments I asked for more detail about the “processing of comments and queries” here. While the authors have attempted to address this comment, I do not think enough detail has been provided. While the authors have now included that “minor changes were made”, I would prefer slightly more detail here. Could the authors provide a sentence here to summarise the changes that were made?
   **Response:** Also in line with comment #15 of reviewer 1, we have clarified now that these minor comments, did not change the content of the summaries [line: 173-175]: *“Comments or corrections from participants on these summaries, which involved minor changes only, were processed and did not change the content of the summaries.”*
10. Line 188: Number 1-10 should be written out i.e. “Phase One...Phase Two”. This needs to be corrected throughout the manuscript.
    **Response:**  Thank you for pointing this out. We have written out the numbers 1-10, throughout the manuscript.

Results

11. Line 210: Throughout the results section, where a quote is presented within a quote there are inconsistencies in the type of quotation marks used (e.g. differences between quotes presented on line 268 and line 495). I suggested checking all quotes throughout the manuscript to standardise the quotation marks used.
    **Response:** Thank you for pointing this out. We have now used single quotations within every quote in which a quote is made.
12. Line 211: Throughout the methods section the authors refer to “two main themes and one extra separate theme”, is there a reason this is not simply a third theme? Furthermore, the results are presented in a different order to the way the methods are presented. Methods are presented two main themes and then “extra theme”, whereas the results section starts with the “extra theme”. I suggest either reordering either the methods or results so that the themes are presented consistently throughout the paper.
    **Response:** This extra separate theme is not a major theme because it has only two layers within the hierarchy describing it (subthemes and main codes), opposed to the three layers of hierarchy (themes, subthemes and main codes) within the two identified major themes. This is also visible in the figures presenting the thematic maps of the theme and the two major themes. We have now added a description to the method section and altered the order of this section so it reflects the structure of the results section. This now reads as follows [line: 189-197]: *“Within the coding tree one extra separate theme “contextual and individual factors” with subthemes and main codes was identified. Additionally, two major themes, including themes, subthemes and main codes were reviewed and identified: 1) reasons for use and non-use of the standing option and 2) strategies to increase the use of the standing option and physical activity at the workplace. This was again discussed within the whole research team and the hierarchy of the extra separate theme and the two major themes were set in thematic maps.”*

Furthermore, in the Results section, we now refer to the thematic maps, as identified in the last paragraph of the method section [line: 232-233]: *“The thematic map with subthemes and main codes of the extra separate theme “contextual and individual factors” are presented in figure 1,*

13. Line 340: “Rationales behind putting the workstation up included to be at the same level as the visitor, shorter meetings and more intense conversations while being standing”. This sentence is quite long and difficult to follow. Could it be simplified? Also I am unsure what “shorter meetings” means? Does that mean that the standing desks can be used for short meetings, or the use of the standing desk shortens the length of a meeting and therefore this is a benefit? Also what does “more intense conversations while being standing mean”, what is an intense conversation? I think this description needs to be reconsidered.
    **Response:** The ‘shorter meetings’ indeed refer to shorter length of meetings, which we have now indicated in the manuscript. More intense conversation is reflecting the quote from the participants directly below this section, in which it was mentioned as the dynamics of the conversations. (“I only had a meeting maybe six weeks ago where we stood around, it was one of the few occasions. There was only five of us so you can stand around these smaller tables quite easily and yes it actually brings people closer together ‘cause you are leaning in and I think the dynamics of the conversation goes a little bit differently rather than sitting down in a line.”). Because this is also mentioned in the quote and to increase readability, we have deleted this terminology from the sentence. This sentence now reads as follows [line 333-335]: *“Rationales behind putting the workstation up included to be at the same level as the visitor and shortening the meeting.”*
14. Line 358: The use of the phrase “switch back” should be reconsidered throughout the manuscript as per my previous review comments as this is an example of colloquial language.
    **Response:** We are sorry to have missed this during the previous revision. We have now changed this phrasing throughout the manuscript into: ‘return to a seated position’, as suggested by the reviewer during the previous revision.
15. Line 367: Incorrect grammar here, rephrase “more easy” to “easier”.
    **Response:** Thank you for pointing this out. We have adjusted this accordingly.
16. Line 374: Should this read “Active alternatives to sedentary activities”? The ending of this sentence as it is currently written leaves me wondering “active alternatives to what”?
    **Response:** Thank you for this suggestion to make this more clear. We have incorporated the suggestion of the reviewer [line 366]: *“…when they chose active alternatives to sedentary activities.”*
17. Line 393: Should this read “as a potential reason” rather than “as potential reason”
    **Response:** We have included ‘a’ in this sentence now, thank you.
18. Line 436: I agree with the authors here that it is best to not change participant quotes as much as possible. To indicate that a change has been made to the participant quote here, I think the following quote should be written like this:
    “it can be promptious [sic, a prompt] seeing a colleague with their desk up and so I think ‘I have not done this for a while’.”
    **Response:** Thank you for this suggestion, we have adjusted the quotation as
    recommended.
19. Discussion
    Line 516: Is there any other evidence to indicate that the use of standing desks improves concentration/ alertness which could be cited here?
    **Response:** To our knowledge, there is no other qualitative research in which this is mentioned specifically related to the use of sit-stand workstation. We think this is primarily because previous research almost exclusively included participants who had short-term access to sit-stand workstations and the information provided in these studies focussed more on the novelty of the furniture, whereas our study included participants with long-term access.
20. Line 519: I suggest rephrasing this sentence to something like “users identified they preferred to use the standing desk when completing tasks which did not require high levels of concentration, such as sending emails”.
    **Response:** Thank you for this suggestion. We have adjusted this sentence, which now reads as follows [line: 504-507]: *“Users indicated they preferred to use the standing workstation when completing work tasks which did not require high levels of concentration, such as sending emails.”*
21. Line 544: The sentence beginning “Still, high-focus” is particularly long and difficult to follow. Could this sentence be simplified or split into two sentences?
    **Response:** We have simplified the sentence and split into two. It now reads [line 528-531]: *“Still, work tasks which required high focus (e.g. typing), were mentioned being preferably done while sitting. This might indirectly mirror a decrease in perceived productivity if these tasks were done standing.”*
