# Supplementary material for: The user and non-user perspective: Experiences of office workers with long-term access to sit-stand workstations
Source: PLoS One. 2020 Jul 28;15(7):e0236582. doi: 10.1371/journal.pone.0236582 (PMC7386596; doi:10.1371/journal.pone.0236582)
Supplement: Attachment — Submitted filename: plos 1.docx [file pone.0236582.s009.docx]

Thanks for the opportunity to re-review. This paper has improved significantly. My main concern is still revolving around the thematic analysis conducted and the reporting of this. I am still quite unclear how the themes were generated, the differences between main themes and subthemes for example. I find the figures explaining the trees don’t enhance the text as they are quite busy and hard to follow in places. I also have concerns around the reporting of saturation. Please see other papers where thematic analysis was used. This may help guide reporting. I feel this would also benefit from a thorough proof read from a native English speaker, ideally who has an understanding of scientific writing.

There are also some specific comments I feel need to be addressed as outlined below:

Line 40: suggest change “because” to “as”

Line 64: I feel by just saying that an increased reach could be yielded, you are underselling the impact. Please reconsider how else it may positively impact. Something along the lines of line 107-108

Line 67: Is the second comma required here?

Line70-72- word sitting used x 3, please re word for clarity

Line 76: Unsure what is meant by “not reducing long-term health risks”, please re word

Line 77-80: consider breaking in to 2 sentences for better readability

Line 91: Unsure who is being referred to in “as the users”- which users?

Line 92: Word perspective used x 2, consider another term eg, view. Also practitioners used x 2 in this sentence.

Line 92-93: Both practitioner and managers are mentioned, but only results for practitioners is explained in the end of the sentence? Does this mean the managers were not informed?

Line 100: Please check if this “n=” needs to be capitalised?

Line 103: Perhaps change to “This current study”

Line 104: Insight into?

Line 115: To keep the tense consistent, should this read had had, and not have had?

Line 117: unsure what small centred means here?

Line 125: Since *user* is pronounced as starting with a consonant "y" sound, the article *a* is appropriate, and *an* is not.

Line 145: consider changing group interviews assisted to “group interviews, assisted”

Line 146:How many were familiar?

Line 163-164: This warm-up exercise for participants was intended to become familiar with the

topic and the group. This sentence does not read well. Perhaps something like “The purpose of the warm-up exercise was to allow participants become familiar with the topic and the group”.

Line 166-169: What was the purpose of doing this if changes were not made? I am struggling to see the relevance of this section, please clarify.

Line 183-187: I cannot understand how these themes were generated, please clarify. Confused re extra and main themes. Because I don’t fully grasp this section, interpreting the results is difficult

Line 183-191: Please consider revising this. Please read other papers where thematic analysis is used and how they report this. Saturation section also needs to be reworked.

Line 205: Table?

Line 206: to participate, consider changing to “in participating”

Line 207:Were the dates/times pre scheduled , or why may some have been unavailable?

Line 209: had long working experience, please reword to something like “long term employees”

Line 211: indicated to use, please change to something like “indicated using it”

Line 221: Capitalise “figure”? Please check is this is required and if so change “table” and “figure” in text throughout.

Line 255-256: Perhaps break into 2 sentences or rephrase- hard to follow

Line 257-259: any reason for this? Why some had a pattern and some preferred to use randomly?

Line 274: practical issues of the workplace- should this read “practical workplace issues”?

Line 274-7: Please reword this, or at least use better punctuation to ensure the reader doesn’t get confused by the “” s and theme names etc

283-285: Please re phrase or break into 2 sentences for clarity and readability

338-9: Should this last “and” be “or”?

416-7: Larger desk surfaces to adjust to standing height- please clarify what is meant here

424-6: Please re phrase or break into 2 sentences for clarity and readability

Line 439: Within the theme individual adjustments, If this read Within the theme “Individual Adjustments”,, it may help readability, please change where this occurs with other themes throughout paper.

442: What is a facility room?

444: Please consider changing comfortable to, to “comfortable for”

454: However, also other emphasis were made during these group interviews. This doesn’t make sense to me. Please rephrase

553-4: in which the organisational culture promotes health behaviour- should this be behaviours? Or what behaviour?

581: consider changing personal room to private office?
