# Supplementary material for: The user and non-user perspective: Experiences of office workers with long-term access to sit-stand workstations
Source: PLoS One. 2020 Jul 28;15(7):e0236582. doi: 10.1371/journal.pone.0236582 (PMC7386596; doi:10.1371/journal.pone.0236582)
Supplement: Attachment — Submitted filename: Response to reviewer.docx [file pone.0236582.s010.docx]

Dear editor,

We feel great effort has been made again by the reviewers, and we have addressed all comments in our point-by-point response here below. Furthermore, we have send our manuscript to a native speaker (with expertise in qualitative research), who has made valuable text and syntax edits. In the revised manuscript this has led to two authors making tracked changes (the native speaker and the corresponding author). We feel this last round of revisions and editing has majorly improved the manuscript. In our response we refer to the line numbers of the manuscript with tracked changes, but for clarity we have cited the (unmarked) text from the final manuscript in this response letter.

Reviewer #1

Thanks for the opportunity to re-review. This paper has improved significantly. My main concern is still revolving around the thematic analysis conducted and the reporting of this. I am still quite unclear how the themes were generated, the differences between main themes and subthemes for example. I find the figures explaining the trees don’t enhance the text as they are quite busy and hard to follow in places. I also have concerns around the reporting of saturation. Please see other papers where thematic analysis was used. This may help guide reporting. I feel this would also benefit from a thorough proof read from a native English speaker, ideally who has an understanding of scientific writing.

**Response:** Thank you for the comments. We have addressed all comments here below and we have had the manuscript checked by a native English speaking editor specialised in qualitative research. The reporting of the thematic analysis, the themes and subthemes and the reporting on the saturation have been altered in response to reviewer two (previous round). We are sorry to find out this has caused confusion. We have clarified the paragraph about the data analysis further, based on the specific comments made by the reviewer here below and by edits from the native speaker. We feel this has further improved the paragraph on data analysis.

There are also some specific comments I feel need to be addressed as outlined below:

1. Line 40: suggest change “because” to “as”
   **Response:** We have adjusted this as suggested by the reviewer [line: 40-41]: *“Recommendations within guidelines for physical activity include the general advice to sit less throughout the day [1-4], as prolonged…”*
2. Line 64: I feel by just saying that an increased reach could be yielded, you are underselling the impact. Please reconsider how else it may positively impact. Something along the lines of line 107-108
   **Response:** Thank you for pointing this out. We have increased the impact by stating [line 66-67]: *“To increase the quality and thus the reach of interventions,…”*
3. Line 67: Is the second comma required here?
   **Response:** We have adjusted this as suggested by the reviewer and in agreement with the editing from the native speaker [line:69-70]: *“To examine this, qualitative research must be conducted which aims to explore and better understand…”*
4. Line70-72- word sitting used x 3, please re word for clarity
   **Response:** Thank you for pointing this out. We have overlooked this during the last round of revisions. We have now rewritten this sentence into: [line 73-75]: *“A recent synthesis of qualitative research [27] included studies among sitting-based office workers which focussed on associated health problems [28] and possible strategies for reducing occupational sitting [29, 30].”*
5. Line 76: Unsure what is meant by “not reducing long-term health risks”, please re word

**Response:** We have changed this sentence now as follows [line 78-80]: *“According to these workers, the most important motivation to reduce sitting time was to enhance musculoskeletal health rather than to reduce long-term health risks, such as diabetes or cardiovascular disease [28].”*

6. Line 77-80: consider breaking in to 2 sentences for better readability
   **Response:** We have now, also in agreement with the native speaker, adjusted it as follows [line: 80-84]: *“ The synthesis also included qualitative studies conducted shortly after the implementation of sit-stand workstations and, focussing on user-experiences with the furniture [31-33], identified support from co-workers and managers to be an overall facilitator. A predominant sitting culture, meanwhile, was determined to be an overall barrier to reducing occupational sitting time.”*
7. Line 91: Unsure who is being referred to in “as the users”- which users?
   **Response:** We have now clarified that this refers to the “current” users included in the referenced study [line: 96-97]: *“…indicating similar levels of enthusiasm as the current users.*
8. Line 92: Word perspective used x 2, consider another term eg, view. Also practitioners used x 2 in this sentence.
   **Response:** Thank you for pointing this out. We have adjusted this as follows [line 92]: *“Other perspectives under study included views of managers and occupational health practitioners, which showed that they were well- informed about the health risks of sitting”*
9. Line 92-93: Both practitioner and managers are mentioned, but only results for practitioners is explained in the end of the sentence? Does this mean the managers were not informed?

**Response:** Thank you for pointing this out, we have clarified now that this reflects on both managers and practitioners. Please see our response to your comment #8.

Line 100: Please check if this “n=” needs to be capitalised?
**Response:** Thank you for pointing this out. As we refer to one population it is not needed to capitalise N. We have adjusted it accordingly into “n=”

10. Line 103: Perhaps change to “This current study”
    **Response:** Thank you , this indeed adds clarity [line 109-111]: *“The aim of the current study was to explore the reasons for use and non-use of sit-stand workstations, as well as to explore other strategies to decrease sitting and increase physical activity in the workplace.”*
11. Line 104: Insight into?
    **Response:** We have adjusted this according to comment #1 of reviewer 2 into explore instead of to gain insight. Please see the answer to comment #10 here above.
12. Line 115: To keep the tense consistent, should this read had had, and not have had?
    **Response:** We have kept the tense consistent now and altered have had into “had had”.
13. Line 117: unsure what small centred means here?
    **Response:** We have clarified now that the part that could be placed in standing position was a small part in the centre of the workstation [line 124-125]: *“Employees had personal, large workstations, with a small, centred compartment adjustable to standing height.”*
14. Line 125: Since *user* is pronounced as starting with a consonant "y" sound, the article *a* is appropriate, and *an* is not.
    **Response:** Thank you for pointing this out, we have adjusted this accordingly into “a user profile”.
15. Line 145: consider changing group interviews assisted to “group interviews, assisted”
    **Response:** We have adjusted this accordingly, by adding the coma.
16. Line 146:How many were familiar?
    **Response:** As the information to identify participants (personal identification data) from the previous study were already removed from the research folders in line with the privacy regulations, this could not be specified anymore. However, according to the researchers’ memory, it were three or four participants.
17. Line 163-164: This warm-up exercise for participants was intended to become familiar with the topic and the group. This sentence does not read well. Perhaps something like “The purpose of the warm-up exercise was to allow participants become familiar with the topic and the group”.
    **Response:** Thank you for this suggestion, we have adjusted this sentence according to the suggestion [line 174-175]: *“The purpose of the warm-up exercise was to allow participants become familiar with the topic and the group.”*
18. Line 166-169: What was the purpose of doing this if changes were not made? I am struggling to see the relevance of this section, please clarify.
    **Response:** We checked if the summaries reflected the opinion of the participants, which seemed to be the case (hence no substantial changes had to be made). After editing of the native speaker, this now reads [line 179-182]: *“Comments or corrections from participants on these summaries, were then processed. The comments indicated minor changes only and did not change the content of the summaries.”*

Furthermore, the first step in the data analysis was to become familiar with the data, which included reading the summaries, which were approved by the participants. We refer to the reading of the summaries in the next paragraph [line 173-174]: *“Phase one involved reading the summaries and transcripts* *in their entirety to become familiar with the data.”*

19. Line 183-187: I cannot understand how these themes were generated, please clarify. Confused re extra and main themes. Because I don’t fully grasp this section, interpreting the results is difficult
    **Response:** We have clarified this section based on earlier comments from reviewer two (previous rounds). This paragraph now includes six steps of thematic coding which we mention explicitly. However, the process in which the actual themes emerged from the initial coding tree, could indeed be more clear. We have added the following explanation, and clarified the grammar now [line: 191-200]: *“During phase three, which encompassed searching for themes, general codes between LR and both coders (MH and ES) were discussed. When differences occurred, consensus between the coders was sought, which resulted in an initial coding tree. Additional codes from LR’s coding of the transcripts were discussed with MH and consensus was again sought. In phases four and five, which encompassed reviewing and naming themes, the initial coding tree was reviewed by LR and HRP, and codes were reordered into potential themes. As a result, one additional separate theme— “Contextual and individual factors”— with subthemes and main codes was identified at this stage. Furthermore, two major themes (including themes, subthemes and main codes,) were identified:…*”
20. Line 183-191: Please consider revising this. Please read other papers where thematic analysis is used and how they report this. Saturation section also needs to be reworked.
    **Response:** Please see our response on comment #19. Considering the remark about saturation. We have indicated now, that saturation is always subject to the researchers interpretation. We hope this is a satisfying answer for the reviewer. [line 191-193]: *“Saturation seemed to only be established for one of the two identified major themes; no new codes which considerably altered the coding tree were identified during coding of the final transcript, although this was subject to the interpretation of the researchers [39].*”
21. Line 205: Table?
    **Response:** Thank you for pointing this out. We have now capitalised Table and Figure (see comment #26) throughout the manuscript.
22. Line 206: to participate, consider changing to “in participating”
    **Response:** Thank you for pointing this out, we have adjusted this according to the reviewers suggestion.
23. Line 207:Were the dates/times pre scheduled , or why may some have been unavailable?
    **Response:** As the participants were all volunteering during working hours, they often had to prioritise their work before participation, although we tried to find consensus on a time which was suitable for all potential participants per group interview.
24. Line 209: had long working experience, please reword to something like “long term employees”
    **Response:** Thank you for this suggestion. We have adjusted the sentence accordingly [line 225-226]: *“In general, participants were highly educated, long-term employees at the organisation who worked full-time.”*
25. Line 211: indicated to use, please change to something like “indicated using it”
    **Response:** We have adjusted this as suggested into “indicated using it”
26. Line 221: Capitalise “figure”? Please check is this is required and if so change “table” and “figure” in text throughout.
    **Response:** We have adjusted this and capitalised “Figure” and “Table” throughout the manuscript.
27. Line 255-256: Perhaps break into 2 sentences or rephrase- hard to follow
    **Response:** We have adjusted the phrasing of this sentence in order to increase the flow. It now reads [line: 258-259]: *“Individual attitudes and behaviours included personal patterns of sitting, standing, and physical activity as well as attitudes towards the health risks of prolonged sitting and standing.”*
28. Line 257-259: any reason for this? Why some had a pattern and some preferred to use randomly?
    **Response:** Users indicated no specific reasons why they used it in a structured manner or not. Users who made use of the standing option more randomly, indicated to use it when they felt like it. We have now included this explanation to the manuscript [line: 275-277]: *“Some indicated that they used it with a clear pattern when switching from standing to sitting, while others used it randomly, when they felt like it, without a specific pattern.”*
29. Line 274: practical issues of the workplace- should this read “practical workplace issues”?
    **Response:** We have adjusted this as suggested by the reviewer into “practical workplace issues.
30. Line 274-7: Please reword this, or at least use better punctuation to ensure the reader doesn’t get confused by the “” s and theme names etc
    **Response:** We have reworded this section to make clear that the theme within “” encompasses the subthemes. This now reads as follows [line 294-297]: *“The theme “Reasons for both use and non-use” included the subthemes work content determines work posture, and experiences and behaviour. The theme “Reasons for use” included the subthemes health benefits and immediate benefits.”*
31. 283-285: Please re phrase or break into 2 sentences for clarity and readability
    **Response:** To add clarity, we have broken the sentence into two [line: 303-305]: *“Among users of the sit-stand workstations a main reason for non-use seemed to be that a persistent habit of sitting prevented them from using the standing option more frequently. Furthermore, users indicated that they tended to forget to use the standing option.”*
32. 338-9: Should this last “and” be “or”?
    **Response:** This would have been an option. However, in this specific case, the reason to return to a seated position (because of tiredness), was indicated by the users as a reason for non-use. So they indicated this as non-use and a reason to return to sitting.
33. 416-7: Larger desk surfaces to adjust to standing height- please clarify what is meant here
    **Response:** This is referring to the small part of the workstations which is adjustable to standing height (mentioned in the methods section in comment #13). Furthermore, we explain this again in the results section before, in which practical issues were reported as part of the theme reasons for non-use [line 304-305]: *“…or because the desk was too full, since only a small part of the workstation surface is adjustable to standing height.”*
34. 424-6: Please re phrase or break into 2 sentences for clarity and readability
    **Response:** To add clarity, we have made two sentences of this phrase, which now reads as follows [line: 451-454]: *“Within organisational adjustments, managers and internal ergo-coaches acknowledged that they could be role models for employees. For instance, they could serve as examples and provide information about the (health) benefits of using the standing option and being less sedentary at the office.”*
35. Line 439: Within the theme individual adjustments, If this read Within the theme “Individual Adjustments”,, it may help readability, please change where this occurs with other themes throughout paper.
    **Response:** Thank you for pointing this out. We have changed this accordingly into “Individual adjustments” and have altered this punctuation to address themes throughout the manuscript.
36. 442: What is a facility room?
    **Response:** This was the phrasing used by the participants. To clarify what they meant we have now added a description [line 469-470]: *“It was suggested that a facility room (i.e. a sort of library) could be set-up, where interested employees could try new tools such as desk bikes.”*
37. 444: Please consider changing comfortable to, to “comfortable for”
    **Response:** We have adjusted this accordingly into “comfortable for them”
38. 454: However, also other emphasis were made during these group interviews. This doesn’t make sense to me. Please rephrase
    **Response:** We have adjusted this sentence to add clarity [line 482-484]: *“However, other topics were also raised during group interviews with managers and ergo-coaches.”*
39. 553-4: in which the organisational culture promotes health behaviour- should this be behaviours? Or what behaviour?
    **Response:** This refers indeed to healthy behaviours, we have adjusted it as suggested by the reviewer [line 588-589]: *“…in which organisational culture promotes healthy behaviours*.”
40. 581: consider changing personal room to private office?
    **Response:** Thank you for this suggestion. We have changed this to private office [line 584-585]: *“…this population was very highly educated, had private offices with personal sit-stand workstations..”*

Reviewer #2:

Thank you for updating the manuscript based on my previous comments. The manuscript is now much improved and will make an excellent contribution to the evidence base for sedentary behaviour interventions in the workplace. I have noted a few minor typographical errors below, however I do not feel the need to review this paper again, when these changes have been made, as they are very minor.

1. I suggest changing wording of the aim of the paper from “gain insight” to “explore”. This will need to be updated on lines 13, 103, 106 and 590.
   **Response:** Thank you for this suggestion. We have adjusted the phrasing in all lines, as suggested by the reviewer.
2. Line 194- full stop at the start of the sentence needs to be removed
   **Response:** This was indeed a mistake and had to be removed, sorry to have missed this mistake during the last round of revisions.
3. Line 108- change = to ± for standard deviation
   **Response:** Instead of standard deviation (SD) we have now used ±.
4. Line 237- should “prove” be “proof”?
   **Response:** Instead of ‘prove’, this indeed needed to be written as ‘proof.’ We have now altered this quote accordingly.
5. Line 472- formatting of quote within a quote needs to be corrected
   **Response:** Thank you for pointing this out. We have altered the quotation within the quote an used single quotations, to be consistent with other within quotations throughout the manuscript.
